# COSMIN systematic review and meta-analysis of the measurement properties of the Positive and Negative Syndrome Scale (PANSS)

**DOI:** 10.1016/j.eclinm.2025.103155

**Published:** 2025-04-11

**Authors:** Simon Geck, Maximilian Roithmeier, Markus Bühner, Sophia Wehr, Lucia Weigel, Josef Priller, John M. Davis, Stefan Leucht

**Affiliations:** a Department of Psychiatry and Psychotherapy, School of Medicine and Health, Technical University of Munich, Klinikum Rechts der Isar, Ismaningerstraße 22, Munich, 81675, Germany; b Department of Psychology, Ludwig Maximilian University of Munich, Leopoldstr. 13, Munich, 80802, Germany; c German Center for Mental Health (DZPG), Site Munich/Augsburg, Germany; d Neuropsychiatry and Laboratory of Molecular Psychiatry, Charité – Universitätsmedizin Berlin and DZNE, Berlin, Germany; e Centre for Clinical Brain Sciences, UK Dementia Research Institute at the University of Edinburgh, Edinburgh, UK; f Psychiatric Institute, University of Illinois at Chicago (mc 912), 1601 W. Taylor St., Chicago, IL, 60612, USA

**Keywords:** Schizophrenia, Antipsychotic drug trials, Positive and Negative Syndrome Scale, PANSS, Psychometrics, Psychotic disorders, Rating scales, Psychopathology

## Abstract

**Background:**

The Positive and Negative Syndrome Scale (PANSS) is the most widely used tool for assessing the symptoms of schizophrenia. Despite its widespread use, the psychometric properties of the PANSS have not been systematically reviewed. This study fills that gap in the scientific literature.

**Methods:**

We utilized the COnsensus-based Standards for the selection of health Measurement INstruments (COSMIN) guideline for systematic reviews and meta-analytical procedures to assess the psychometric properties of the PANSS in its original three-subscale form as well as the quality level of the evidence available. On this basis we formulated recommendations for future research and use. A study protocol was registered under 10.17605/OSF.IO/5EGMD. The search period was until February 21, 2024.

**Findings:**

We included 119 publications. According to COSMIN, the PANSS demonstrated sufficient reliability, construct validity, and responsiveness; but had significant shortcomings in content validity and structural validity. The original three-factor model showed poor structural validity, leading to its COSMIN classification as “not recommendable”. The subscales showed overall acceptable measurement properties. However, the lack of structural validity of the three-subscale model renders its subscales less useful. Moreover, the PANSS negative subscale does not cover all domains of the National Institute of Mental Health consensus. Due to the length of the instrument (30–50 min), it is barely useable in clinical practice.

**Interpretation:**

Although the PANSS is the standard scale for schizophrenia symptom severity, its shortcomings regarding fundamental psychometric domains and practical applicability warrant the development of new scales for which appropriate methods should be applied from the start.

**Funding:**

There was no specific funding source for this research.

**Research in context**

**Evidence before this study**

The Positive and Negative Syndrome Scale (PANSS) is probably the most frequently used rating scale for assessing the symptoms of schizophrenia. Therefore, it is necessary to systematically review the measurement properties of the PANSS.

We therefore performed a search for reviews on the psychometric properties of the PANSS via PubMed. In 2015, Garcia-Portilla et al. conducted a systematic review on the measurement properties of the PANSS negative subscale but addressed neither the total PANSS nor the positive and general psychopathology subscale and further did not follow a structured and comprehensive approach. To date, no systematic review covering the full range of available data regarding the measurement properties of the whole PANSS scale and its three subscales was published. We fill this gap by applying the COnsensus-based Standards for the selection of health Measurement INstruments (COSMIN) guidelines for systematic reviews of patient-reported outcome measures.

**Added value of this study**

We present the first systematic review and meta-analysis of the psychometric properties of the PANSS. Without language restrictions, we searched the databases MEDLINE and Embase for evaluation studies of the PANSS published between the initial publication of the PANSS and February 2024 and identified 119 publications containing the assessment of 10 different psychometric properties. Quantitative results were meta-analytically pooled and no indications for small-trial bias have been found. We found that the PANSS has strengths in terms of reliability, construct validity, and responsiveness. There are weaknesses in the domains content validity and structural validity which are considered fundamental by COSMIN. The feasibility of the scale in practice is questionable. Research suggests a 5-factor structure rather than the original 3 subscale form.

**Implications of all the available evidence**

The PANSS has been successfully applied to separate between interventions in schizophrenia trials. Nevertheless, given its shortcomings in terms of content validity, structural validity, lack of covering important domains of negative symptoms and feasibility, the development with modern methods of a new scale to rate symptoms of schizophrenia is warranted. Moreover, we recommend further research into five-factor solutions of the PANSS and short versions.

## Introduction

The Positive and Negative Syndrome Scale (PANSS) is the most frequently used scale to rate the symptoms of schizophrenia.^1^ It was developed in the 1980s by Kay et al. when it became clear that negative symptoms are at least as significant, if not more significant, than the positive symptoms of schizophrenia.^1^^,^^2^ For example, Crow had hypothesized that there is a type I schizophrenia characterized by positive symptoms and good response to antipsychotic drugs, and a type II schizophrenia predominated by negative symptoms and associated with poorer outcome.^3^^,^^4^ Similarly, Andreasen coined the terms positive and negative schizophrenia.^2^ In practice the acute phase is often dominated by positive symptoms, while negative symptoms develop later, and some of these negative symptoms may be the result of extrapyramidal side-effects. At that time, the most commonly used 18-item version of the Brief Psychiatric Rating Scale (BPRS) did not sufficiently cover negative symptoms.^2^ Therefore, a new scale had to be developed. Following the two-dimensional positive versus negative symptoms illness concept and adding a third domain for measuring general psychopathology, the PANSS was created.^2^^,^^5^ It is a synthesis of all 18 items of the BPRS with 12 items from the 32-item Psychopathology Rating Schedule (PRS), which Kay and colleagues had put together in the 1970s without, however, following valid scale-development methods.^6^^,^^7^ In order to improve on the BPRS, the severity levels of each of the 30 PANSS items were anchored by clear descriptions.^1^ Thus, the PANSS consists of 3 subscales (positive scale, negative scale, and general psychopathology scale) with 7 items each on the positive and the negative subscale, and 16 items on the general psychopathology subscale, all rated between 1 “absent” and 7 “extreme”.^1^

Despite the predominance of the PANSS in rating symptoms of schizophrenia, its psychometric properties have, to the best of our knowledge, never been addressed by a systematic review and meta-analysis. We fill this gap by applying the COnsensus-based Standards for the selection of health Measurement INstruments (COSMIN) guideline, which was developed in an international Delphi study to evaluate the measurement properties of health-related patient-reported outcomes.^8^, ^9^, ^10^ In the current publication, we addressed the total score and the original three PANSS subscales. In companion publications, we review various factor solutions (e.g., the 5-factor solution found by Marder et al. 1997), and short-versions such as PANSS-6.^11^^,^^12^

## Methods

**Fig. 1:**
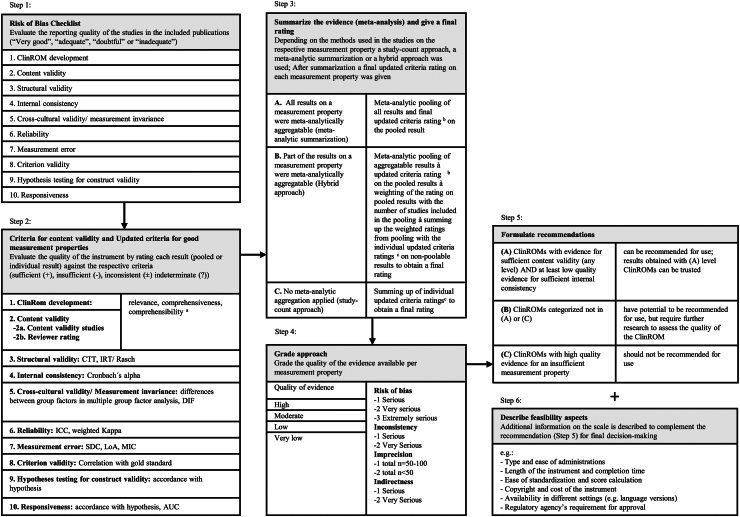
**Overview of the COSMIN methodology including adaptions**. Refer to the methods part and appendix of our publication for further information. For the unmodified, full version of the COSMIN methodology, refer to the COSMIN manual. Step 1: Risk of Bias Checklist. Step 2: *Criteria for content validity* and *Updated criteria* for good measurement properties. ^a^For the detailed criteria refer to the COSMIN manual for content validity. Step 3: Summarize the evidence and give a final rating. ^b^The same criteria like in Step 2 apply. ^c^These are the individual ratings on each result obtained in Step 2. Step 4: Grade approach. Step 5: Formulate recommendations. Step 6: Describe feasibility aspects.

The methods used in this review are based on the COSMIN guideline for systematic reviews of patient-reported outcome-measures.^8^, ^9^, ^10^ COSMIN was initially created for patient-reported outcome-measures (PROMs) but can also be applied to clinician-reported outcome-measures (ClinROMs), such as the PANSS.^13^^,^^14^ There are several steps: *literature search*, quality assessment of the individual studies with the COSMIN *Risk-of-Bias checklist*, applying *updated criteria for good measurement properties*, *summarizing the evidence*, *grading the overall quality of evidence* with a modified Grading of Recommendations Assessment, Development and Evaluation (GRADE) approach, formulating a *recommendation* and describing *feasibility aspects*.^8^, ^9^, ^10^ An overview is presented in Fig. 1.

All steps of the COSMIN methodology were conducted independently by two reviewers (SG and MR). Any disagreements were resolved by consensus, with the involvement of a third reviewer (SL, MB), or the COSMIN group (WM) if necessary.

A protocol was registered on the Open-Science-Foundation website where more details can be found (https://doi.org/10.17605/OSF.IO/5EGMD; compare Appendix S1).^15^

### Search strategy and selection criteria

A PhD librarian (FS) searched PubMed and EMBASE using a modified COSMIN filter, see Appendix S2 and Terwee et al. (2009), for journal articles published until February 21, 2024.^16^ We screened the results and reference lists of included publications for evaluation studies of the PANSS in which at least 80% of study participants had a psychotic disorder in any stage (e.g., acute or in remission). There were no language or country restrictions. Following COSMIN, only full-text articles were included.^8^, ^9^, ^10^ We addressed the original PANSS total, positive, negative and general scores.^1^

### Assessing the risk of bias (step 1 in Fig. 1)

The COSMIN risk of bias checklist consists of 10 domains (called “boxes” by COSMIN): (1) ClinROM development, (2) content validity, (3) structural validity, (4) internal consistency, (5) cross-cultural validity/measurement invariance, (6) reliability, (7) measurement error, (8) criterion validity, (9) hypothesis testing for construct validity, (10) responsiveness.^8^, ^9^, ^10^ Each box consists of two to eight items rated as either “very good”, “adequate”, “doubtful”, “inadequate”, or “not applicable”. A worst score counts principle is applied for each domain.

For criterion validity, the Brief Psychiatric Rating Scale (BPRS) was considered as the gold-standard because of its reported empirical development, stable factor structure, and good reliability.^6^^,^^17^ Risk of Bias (RoB) within COSMIN addresses whether and how well these domains were assessed. The results (e.g., whether Cronbach's alpha was acceptably high) are part of the next step.

### Assessing the criteria for content validity and the updated criteria for good measurement properties (step 2 in Fig. 1)

The results of each study are evaluated following COSMIN's *criteria for I. content validity* (Fig. 1, step 2, domains 1 and 2) and II. The *updated criteria for good measurement properties*.^8^, ^9^, ^10^ (Fig. 1, step 2, domains 3–10).

A study's evidence for each domain is rated as either “sufficient” (+) or “insufficient” (−), with a third option for an “indeterminate” rating (?), if neither “sufficient” nor “insufficient” ratings are applicable. Only for content validity (domains 1 and 2), a fourth rating option of “inconsistent” (±) is available.

The overall rating for I. *Content validity* is generally made up of three individual ratings for the ClinROM development study, content validity studies, and a reviewer rating of the scale (see Appendix S3).

The II. *Updated criteria* address domains 3–10. Concerning domain 3 “Structural validity”, the COSMIN guideline does not provide criteria for factor models resulting from exploratory methods. After consultation with the COSMIN group, we therefore created criteria based on a modified version of the *Criteria for good measurement properties* used by Elsman et al. (2022), for the purpose of comparing the exploratory derived models to Kays' three-factor model.^18^ The criteria are: 1) ≥50% explained variance, 2) factor loadings >0.30, 3) ≤10% cross-loading items, 4) three-factor model, 5) ≥80% of the items distributed like in the Kay three-factor model. A “sufficient” rating was only given when all of the criteria regarding the exploratory derived models were met. Otherwise, the respective rating was “insufficient”. The criteria for structural validity established by Elsman et al. (2022) are identical for both exploratory factor analyses (EFAs) and principal component analyses (PCAs); hence, we generalized this approach to the entire review.^18^

For assessing domains 9 “construct validity” and 10 “responsiveness”, COSMIN requires a formulation of hypotheses which are presented in Appendix S4.^8^, ^9^, ^10^

We considered correlations of the PANSS total score and the subscores with other scales; correlations of single PANSS items were not considered.

For details about the assessment of all other domains suggested by COSMIN, please see pp.28 and 29 of the COSMIN manual.^8^, ^9^, ^10^

If uncertainties arose regarding results or methods of relevant articles, their authors were contacted and asked to revalidate the results or processes. This applied to 39 articles.

### Statistical method

In situations where few studies are available, a simple study-count approach is sufficient in COSMIN reviews, see e.g., Weigel et al. (2023), Wehr et al. (2024) or Zúñiga Le-Bert et al. (2024).^13^^,^^14^^,^^19^ As our review includes 119 studies, we were able to summarize measures of internal consistency, cross-cultural validity, reliability, criterion validity, and construct validity with standard random-effects meta-analyses using Comprehensive Meta-Analysis (CMA) Version 2.^20^ In situations where it was not possible to aggregate *all* studies, we used a hybrid of the study-count and the meta-analytic approach, i.e., the meta-analytic result weighted by the number of studies included and summed up with ratings of studies that couldn't be aggregated and were therefore rated individually.

We measured heterogeneity with the I^2^-statistic and a chi-square test (compare Appendix S5); and small-trial/publication bias with funnel-plots and Egger's test when at least 10 studies were available. In most studies, several raters assessed multiple study participants. In some studies, multiple raters assessed small sample sizes (n ≤ 4). CMA cannot process sample sizes n ≤ 4.^20^ We included the respective studies by exchanging the rater and patient count. Sensitivity analysis without them showed no important differences, allowing us to include these studies.

### Grading the quality of evidence (step 4 in Fig. 1)

The COSMIN modified GRADE approach applies to the accumulated evidence on each measurement property, spanning 4 domains: (1) risk of bias, (2) inconsistency, (3) imprecision, and (4) indirectness.^8^, ^9^, ^10^

The final quality of the evidence is graded as high, moderate, low, or very low (Table 1a). Initially, evidence is assumed to be of high quality. If the criteria of the domains are not fulfilled, the evidence is then downgraded by one to three levels (see Table 1b).

**Table 1:** GRADE approach.

**a) Quality of evidence definitions**	
High	We are very confident that the true measurement property lies close to that of the estimate of the measurement property
Moderate	We are moderately confident in the measurement property estimate: the true measurement property is likely to be close to the estimate of the measurement property, but there is a possibility that it is substantially different
Low	Our confidence in the measurement property estimate is limited: the true measurement property may be substantially different from the estimate of the measurement property
Very low	We have very little confidence in the measurement property estimate: the true measurement property is likely to be substantially different from the estimate of the measurement property
**b) Instructions on downgrading**	
**Risk of bias**	
No	There are multiple studies of at least adequate quality, or there is one study of very good quality available
1 Serious	There are multiple studies of doubtful quality available, or there is only one study of adequate quality
−2 Very serious	There are multiple studies of inadequate quality, or there is only one study of doubtful quality available
−3 Extremely serious	There is only one study of inadequate quality available
**Inconsistency**	
No	Results could be meta-analytically pooled or ≥75% of the studies had consistent results
−1 Serious	<75% of studies had consistent results
−2 Very serious	<70% and ≥65% had consistent results If <65% had consistent results GRADE will not be performed because there is not enough evidence
**Imprecision**^a^	As recommended by COSMIN due to the high total sample sizes no downgrading for imprecision was done
**Indirectness**	As recommended by COSMIN due to our inclusion criteria of ≥80% population with a psychotic disorder no downgrading for indirectness was done

a Downgrading for Imprecision is not applicable for measurement properties in which the sample size is already evaluated in the “Risk of bias” step (studies on content validity, structural validity and cross-cultural validity/measurement invariance).

### Recommendations (step 5 in Fig. 1)

According to COSMIN, an overall judgment of (A) “can be recommended for use and results obtained with the scale can be trusted”, (B) “potential for recommendation but needs further research into the measurement properties”, or (C) “should not be recommended for use” was made.^8^, ^9^, ^10^

Recommendations are primarily dependent on the evidence for content validity and internal consistency (compare Fig. 1, Step 5).

### Feasibility (step 6 in Fig. 1)

To supplement the recommendations, we compiled information on the feasibility of the PANSS (compare Fig. 1, Step 6).

### Role of the funding source

There was no funding source for this study.

## Results

### Literature search

**Fig. 2:**
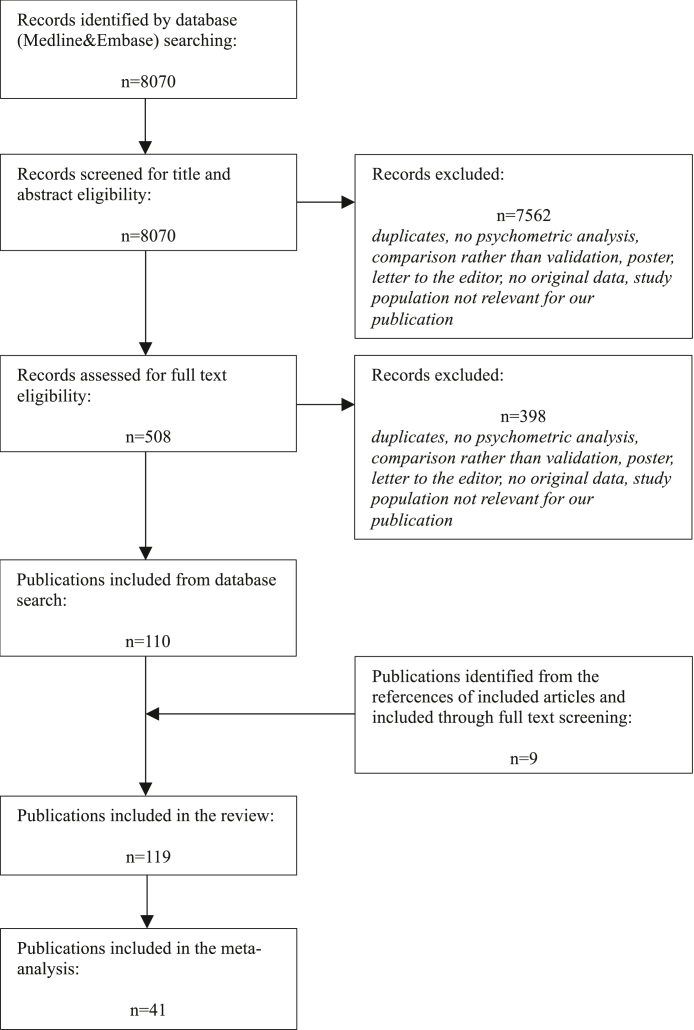
**PRISMA diagram of the search**.

The search yielded 8070 results. Their titles and abstracts were assessed and 508 articles were selected for full-text screening. 110 publications were identified as eligible for this review. The additional screening of references resulted in 9 further inclusions. A flowchart depicting this process can be found in Fig. 2. For the general characteristics of included articles populations, refer to Appendix S6.

### Assessing the risk of bias

Table 2 provides an overview of the distribution of the risk of bias and *Updated criteria* ratings.

**Table 2:** COSMIN risk of bias and criteria results.

Measurement property	Scale (No. of studies assessing the scales measurement property)	COSMIN risk of bias				COSMIN updated criteria ratings^a^			
		Very good	Adequate	Doubtful	Inadequate	+^b^	−c	?^d^	±^e^
ClinROM Development	PANSS total score (n = 1)	0	0	1	0	0	0	0	1
Content validity	PANSS total score (n = 0)	0	0	0	0	0	0	0	0
Structural validity	PANSS total score (n = 134)	28	53	24	28	0	111	22	
	Positive subscale (n = 1)	0	1	0	0	0	0	1	
	Negative subscale (n = 3)	1	2	0	0	0	0	3	
	General psychopathology subscale (n = 1)	0	1	0	0	0	0	1	
Internal consistency	PANSS total score (n = 13)	0	0	0	13	0	0	13	
	Positive subscale (n = 15)	14	0	1	0	0	0	15	
	Negative subscale (n = 16)	15	0	1	0	0	0	16	
	General psychopathology subscale (n = 11)	10	0	1	0	0	0	11	
Cross-cultural validity	PANSS total score (n = 4)	0	0	3	1	0	0	4	
	Positive subscale (n = 6)	0	0	3	3	0	0	6	
	Negative subscale (n = 6)	0	0	3	3	0	0	6	
	General psychopathology subscale (n = 6)	0	0	3	3	0	0	6	
Interrater reliability	PANSS total score (n = 13)	4	6	3	0	13	0	0	
	Positive subscale (n = 18)	4	4	10	0	18	0	0	
	Negative subscale (n = 18)	4	4	10	0	18	0	0	
	General psychopathology subscale (n = 17)	4	3	10	0	17	0	0	
Test-retest reliability	PANSS total score (n = 3)	0	0	2	1	3	0	0	
	Positive subscale (n = 2)	0	0	2	0	2	0	0	
	Negative subscale (n = 2)	0	0	2	0	2	0	0	
	General psychopathology subscale (n = 2)	0	0	2	0	2	0	0	
Measurement error	PANSS total score (n = 4)	0	0	1	3	0	0	4	
	Positive subscale (n = 4)	0	0	1	3	0	0	4	
	Negative subscale (n = 4)	0	0	1	3	0	0	4	
	General psychopathology subscale (n = 4)	0	0	1	3	0	0	4	
Criterion validity	PANSS total score (n = 4)	4	0	0	0	4	0	0	
Hypothesis testing for construct validity	PANSS total score (n = 7)	1	1	5	0	5	2	0	
	Positive subscale (n = 38)	7	24	6	1	36	1	1	
	Negative subscale (n = 40)	7	25	6	2	37	2	1	
	General psychopathology subscale (n = 20)	1	15	2	2	16	4	0	
Responsiveness	PANSS total score (n = 6)	3	1	2	0	6	0	0	

a ClinROM development rating resulted from *Criteria for content validity;* 2 of the 95 (−) exploratory-derived-model-ratings result from Generalized Structured Component Analysis (GSCA) and exploratory structured equation modeling (ESEM), which are not considered in the COSMIN framework. As those studies (models) fail to replicate a three-factor structure (like the original PANSS model), and EFA models are rated (−) as soon as 1 criterion is not fulfilled, those more specific structural equation modeling (SEM) studies are still rated ‘insufficient’.

b “Sufficient”.

c “Insufficient”.

d “Indeterminate”.

e “Inconsistent”.

*ClinROM development* and *content validity* of the PANSS could not be well assessed, because only the original study of the scale provided data and was rated as “doubtful”. In the ClinROM development study of the PANSS, only a synthesis of two existing scales was done.^1^ Thus, the concept elicitation and rational development of the PANSS were not executed in a sufficiently thorough manner.

A large number of studies assessing the *structural validity* of the PANSS total was identified.^5^^,^^11^^,^^12^, ^20^, ^21^, ^22^, ^23^, ^24^, ^25^, ^26^, ^27^, ^28^, ^29^, ^30^, ^31^, ^32^, ^33^, ^34^, ^35^, ^36^, ^37^, ^38^, ^39^, ^40^, ^41^, ^42^, ^43^, ^44^, ^45^, ^46^, ^47^, ^48^, ^49^, ^50^, ^51^, ^52^, ^53^, ^54^, ^55^, ^56^, ^57^, ^58^, ^59^, ^60^, ^61^, ^62^, ^63^, ^64^, ^65^, ^66^, ^67^, ^68^, ^69^, ^70^, ^71^, ^72^, ^73^, ^74^, ^75^, ^76^, ^77^, ^78^, ^79^, ^80^, ^81^, ^82^, ^83^, ^84^, ^85^, ^86^, ^87^, ^88^, ^89^, ^90^ Most of them (60.90%) received “adequate” or “very good” ratings. Only few studies on the structural validity of the subscales were identified.^49^^,^^91^^,^^92^ All of them received “adequate” or “very good” ratings.

All studies on the internal consistency of the PANSS total received “inadequate” ratings, as internal consistency statistics should not be calculated for scores of scales that are not unidimensional or consist of subscores.^21^^,^^29^^,^^37^^,^^74^^,^^80^^,^^93^, ^94^, ^95^, ^96^, ^97^, ^98^ Most studies (90.91%–93.75%) on the internal consistency of the subscales received “very good” ratings.^1^^,^^55^^,^^74^^,^^80^^,^^82^^,^^98^, ^99^, ^100^, ^101^, ^102^, ^103^, ^104^, ^105^, ^106^, ^107^, ^108^

Only few studies on the *cross-cultural validity* of the PANSS total and the three subscales were found and all of them received either “doubtful” or “inadequate” ratings.^109^, ^110^, ^111^, ^112^

The majority (76.92%) of the studies on the *interrater reliability* of the PANSS total received “adequate” or “very good” ratings.^80^^,^^95^^,^^96^^,^^101^^,^^102^^,^^107^^,^^113^, ^114^, ^115^, ^116^ No studies on interrater reliability received “inadequate” ratings, but most of the studies (55.56%–58.82%) on the subscales received “doubtful” ratings.^80^^,^^82^^,^^93^^,^^101^^,^^102^^,^^105^, ^106^, ^107^^,^^109^^,^^110^^,^^113^, ^114^, ^115^^,^^117^

Only few studies on *test-retest reliability* were found and all of them received “doubtful” or “inadequate” ratings.^1^^,^^96^^,^^98^^,^^118^

Only few studies on *measurement error* were found for the PANSS total and its subscales, and all studies received “doubtful” or “inadequate” ratings.^98^^,^^116^

Only few studies on the *criterion validity* of the PANSS total were found.^101^^,^^102^ All of them received “very good” ratings.

Only few studies for *hypothesis testing for construct validity* of the PANSS total were found and the majority (71.43%) of them received “doubtful” ratings.^21^^,^^95^^,^^118^, ^119^, ^120^ Most studies on construct validity were found for the positive and negative subscales. The majority (80.00%–81.58%) of the studies on the construct validity of the subscales received “adequate” or “very good” ratings.^60^^,^^80^^,^^82^^,^^92^^,^^94^, ^95^, ^96^^,^^99^, ^100^, ^101^^,^^103^^,^^105^^,^^107^^,^^117^^,^^118^^,^^121^, ^122^, ^123^, ^124^

Only few studies on the *responsiveness* of the PANSS total were found and none on the responsiveness of the subscales.^29^^,^^120^^,^^125^, ^126^, ^127^ All those studies received “very good” ratings.

More detailed information on the RoB rating process is available from the corresponding author upon request.

### Assessing the criteria for content validity and the updated criteria for good measurement properties

#### Assessing the criteria for content validity

Due to the lack of content validity studies, the content validity rating is based on the rating of the ClinROM development study and the reviewer rating (Table 2).^8^, ^9^, ^10^

The development study of the PANSS and the reviewer rating received “inconsistent” ratings due to weaknesses in *Relevance*, *Comprehensiveness* and *Comprehensibility*.^1^ The overall content validity rating was “inconsistent”.

For a more comprehensive description of this rating process, we refer to Appendix S3.

#### Assessing the updated criteria for good measurement properties

These results are summarized in Table 2.

For the forest-plots and respective 95%-confidence intervals of the results from meta-analysis for the measurement properties *internal consistency*, *interrater-reliability*, and *construct validity*, we refer to Panel 1. For all remaining meta-analytical data, we refer to Appendix S7.

Panel 1 **Results from meta analysis.**

Standard random-effects meta-analyses were conducted using Comprehensive Meta-Analysis Version 2; Panel 1a) Cronbach's alphas for *internal consistency*, Panel 1b) correlation coefficients for *interrater reliability*, Panel 1c) correlation coefficients for *convergent validity*.

##### Internal structure

###### Structural validity

95 exploration-derived models and 16 confirmatory-factor-analyses of the original three-subscale structure were identified.^5^^,^^12^^,^^23^, ^24^, ^25^, ^26^^,^^30^, ^31^, ^32^, ^33^, ^34^, ^35^, ^36^, ^37^, ^38^, ^39^, ^40^, ^41^, ^42^, ^43^, ^44^, ^45^, ^46^, ^47^,^50^, ^51^, ^52^, ^53^, ^54^^,^^56^, ^57^, ^58^, ^59^, ^60^, ^61^, ^62^, ^63^, ^64^, ^65^, ^66^, ^67^, ^68^, ^69^, ^70^,^71^, ^72^, ^73^, ^74^, ^75^, ^76^, ^77^, ^78^, ^79^, ^80^, ^81^, ^82^, ^83^, ^84^, ^85^, ^86^, ^87^, ^88^, ^89^, ^90^^,^^122^^,^^128^, ^129^, ^130^, ^131^, ^132^, ^133^, ^134^ Their assessment could not confirm this original structure resulting in 111 “insufficient” ratings. Confirmatory-fit-indices (comparative fit indices) ranged between 0.517 and 0.920. 22 studies applied Item-Response-Theory/Rasch analyses to the original three subscale structure set up by Kay et al. (1987) which did not fulfill all necessary *Updated criteria* for a (+)/(−) rating and were therefore rated as “indeterminate”.^1^^,^^11^^,^^21^^,^^22^^,^^27^, ^28^, ^29^^,^^38^^,^^48^^,^^49^^,^^55^^,^^135^ Consequently, the overall rating on the structural validity of the original three subscale structure is “insufficient”. 17 of the 22 studies assessed the scalability of the PANSS. 15 studies conclude that the PANSS is not scalable, thus supporting the evidence for insufficiency of the structural validity of the PANSS.^11^^,^^22^^,^^27^, ^28^, ^29^ The lack of scalability of the PANSS, as demonstrated by these 15 studies, essentially refers to its failure to fit the Rasch model.

Four PCAs assessing the dimensionality of the subscales and one Rasch analysis of the negative subscales' scalability were identified.^49^^,^^91^^,^^92^ All five assessments on structural validity of the subscales were rated as “indeterminate” because the assessment methods used are not considered by COSMIN (COSMIN only provides unidimensionality criteria for IRT/Rasch analyses) or because not all criteria required by COSMIN were present (for IRT/Rasch analyses, COSMIN requires 4 criteria to be met: no violation of unidimensionality, of local independence and of monotonicity and adequate model fit for a “sufficient” rating. Only one of them was met). The assessments delivered inconsistent results on the unidimensionality of the negative subscale.^49^^,^^92^ A lack of scalability of the negative subscale and of unidimensionality for the general psychopathology scale were reported.^49^^,^^91^ The unidimensionality assessment of the positive subscale concluded that it is unidimensional.^49^

###### Internal consistency

55 studies on internal consistency were identified.^1^^,^^21^^,^^29^^,^^37^^,^^55^^,^^74^^,^^80^^,^^82^^,^^93^, ^94^, ^95^, ^96^, ^97^, ^98^, ^99^, ^100^, ^101^, ^102^, ^103^, ^104^, ^105^, ^106^, ^107^, ^108^ At least low evidence for sufficient structural validity is a COSMIN requirement for a “sufficient” rating on internal consistency.^8^, ^9^, ^10^ Thus, even though the pooled Cronbach's alpha values of α = 0.730 (positive subscale), α = 0.844 (negative subscale), α = 0.754 (general psychopathology subscale) and α = 0.859 (PANSS total) surpass the threshold of α ≥ 0.70 which is deemed as acceptable by COSMIN and satisfies the common notion of using the threshold of α ≥ 0.70 for an instrument to have an acceptable level of internal consistency, the internal consistency ratings of the original three subscale structure of the PANSS and its subscales were “indeterminate”.^8^, ^9^, ^10^^,^^136^^,^^137^

##### Remaining measurement properties

For the remaining measurement properties (cross-cultural validity, reliability, measurement error, criterion validity, construct validity, and responsiveness) the ratings apply for both the PANSS total and all three subscales, except for criterion validity and responsiveness, for which only evidence on the PANSS total was found.

###### Cross-cultural validity/measurement invariance

22 studies on *cross-cultural validity* could be identified, while none were available on *measurement invariance*.^109^, ^110^, ^111^, ^112^ Percentage differences of least square means from Analysis of Covariance and Pearson correlation coefficients were used to compare the PANSS in American versus Chinese, as well as between Spanish and English PANSS assessments. Percentage differences of least square means (LSM) ranged between 0.1% and 5.1% in one publication and between 8.8% and 37.5% in a second.^111^^,^^112^ Aggarwal et al. ascribe the differences between the Chinese and American samples mainly to cultural differences.^111^^,^^112^ Pooled Pearson correlations of the subscales were strong and ranged between 0.77 and 0.905, indicating satisfactory cross-cultural validity.^109^^,^^110^^,^^138^ Nonetheless, COSMIN requires other tests to assess cross-cultural validity than simple Pearson correlations and percentage differences of LSM. Thus, cross-cultural validity is rated as “indeterminate”.^8^, ^9^, ^10^

###### Measurement error

The identified 16 studies on *measurement error* used G-theory and provided moderate to high generalizability-coefficients between 0.647 and 0.93, indicating that a moderate to large proportion of the variance in the observed scores is due to the true score variance, not due to error variance.^98^^,^^116^^,^^139^^,^^140^ However, G-theory is not a method considered by COSMIN to assess measurement error, which was therefore rated as “indeterminate” as well.^8^, ^9^, ^10^

###### Reliability and criterion validity

The pooled results for studies on interrater reliability (n_studies_ = 66; r_pooled_-range = 0.790–0.885), test-retest reliability (n_studies_ = 9; r_pooled_-range = 0.737–0.797), and criterion validity (n_studies_ = 4; r_pooled_ = 0.877), surpass the *Updated criteria* criterion of r ≥ 0.7, resulting in reliability and criterion validity ratings of “sufficient”.^1^^,^^8^, ^9^, ^10^^,^^80^^,^^82^^,^^93^^,^^95^^,^^96^^,^^98^^,^^101^^,^^102^^,^^105^, ^106^, ^107^^,^^109^^,^^110^^,^^113^, ^114^, ^115^, ^116^, ^117^, ^118^

###### Construct validity

Known groups validity: Two studies assessing the ability of the scales to discriminate between different disease stages were identified.^99^ Analysis of Variance was used, which is not considered by COSMIN, resulting in “indeterminate” ratings, even though the authors report a lack of group differences on control variables, indicating good discriminant validity.^8^, ^9^, ^10^^,^^99^

Convergent and divergent validity: 105 studies correlating PANSS scores to measures of schizophrenia symptom severity, neurocognition, functioning, global assessment, and other more specific measurement scales were identified.^21^^,^^60^^,^^80^^,^^82^^,^^92^^,^^94^, ^95^, ^96^^,^^99^, ^100^, ^101^^,^^103^^,^^105^^,^^107^^,^^117^, ^118^, ^119^, ^120^, ^121^, ^122^, ^123^, ^124^

A sufficient percentage of hypotheses (71.43%–97.30%) was confirmed to rate construct validity as “sufficient”.

###### Responsiveness

As all hypotheses for responsiveness (n_studies_ = 6) were confirmed, responsiveness was rated as “sufficient”.^29^^,^^120^^,^^125^, ^126^, ^127^

##### Publication bias

Internal consistency, interrater reliability, and the correlation between the positive and negative subscale were investigated for small-trial/publication bias, and no indications for such bias were identified.

### GRADE approach

For the PANSS total, the qualities of the evidence on the structural validity, reliability, criterion validity, and responsiveness was graded as “high”, on the cross-cultural validity and construct validity as “moderate” and on the content validity, internal consistency and measurement error as “low”.

The quality of the evidence for the subscales was graded as “high” regarding internal consistency, reliability and construct validity, as “moderate” to “high” regarding structural validity, as “moderate” regarding cross-cultural validity, and as “low” regarding measurement error.

The detailed results on the quality of evidence together with the reasons for downgrading are presented in Table 3.

**Table 3:** COSMIN Summary of findings.

	Summary or pooled result	Overall rating	Quality of evidence
**Content validity**			
PANSS-30 total score	Relevance rating: ±, Comprehensiveness rating: -, Comprehensibility rating: ± (overall ratings resulting from the PROM development study and the reviewer rating)	Inconsistent	Low (no content validity studies available)
**Structural validity**			
Positive subscale	Unidimensional score	Indeterminate	Moderate (just one adequate study available)
Negative subscale	1 out of 2 PCA analyses result in unidimensionality; 1/1 scalability assessments reject scalability	Indeterminate	High
General psychopathology subscale	No unidimensional score	Indeterminate	Moderate (one adequate study available)
PANSS-30 total score	None of the EFA derived models fulfill the criteria; CFI-range: 0.517–0.920 (total sample size: 6114), RMSEA-range: 0.108–0.123 (total sample size: 2644), 15/17 scalability assessments reject scalability	Insufficient	High
**Internal consistency**			
Positive subscale	Pooled: Cronbach's α = 0.730 (total sample size: 1808)	Indeterminate (15× (?))	High
Negative subscale	Pooled: Cronbach's α = 0.844 (total sample size: 1854)	Indeterminate (16× (?))	High
General psychopathology subscale	Pooled: Cronbach's α = 0.754 (total sample size: 10,694)	Indeterminate (11× (?))	High
PANSS-30 total score	Pooled: Cronbach's α = 0.859 (total sample size: 1796)	Indeterminate (13× (?))	Low (only inadequate studies available)
**Cross-cultural validity**			
Positive subscale	Pooled: r = 0.905 (total sample size: 37); percentage difference range: 0.6–37.5%	Indeterminate (6× (?))	Moderate (multiple doubtful studies available)
Negative subscale	Pooled: r = 0.776 (total sample size: 37); percentage difference range: 0.1%–8.8%	Indeterminate (6× (?))	Moderate (multiple doubtful studies available)
General psychopathology subscale	Pooled: r = 0.770 (total sample size: 37); percentage difference range: 5.1–34.3%	Indeterminate (6× (?))	Moderate (multiple doubtful studies available)
PANSS-30 total score	Percentage difference range: 5.1–26.1%	Indeterminate (4× (?))	Moderate (multiple doubtful studies available)
**Reliability**			
Positive subscale	Pooled: interrater reliability: r = 0.885 (total sample size: 596), test-retest reliability: r = 0.791 (total sample size: 122)	Sufficient	High
Negative subscale	Pooled: interrater reliability: r = 0.790 (total sample size: 599), test-retest reliability: r = 0.762 (total sample size: 122)	Sufficient	High
General psychopathology subscale	Pooled: interrater reliability: r = 0.828 (total sample size: 514), test-retest reliability: r = 0.737 (total sample size: 122)	Sufficient	High
PANSS-30 total score	Pooled: interrater reliability: r = 0.812 (total sample size: 701), test-retest reliability: r = 0.797 (total sample size: 1771)	Sufficient	High
**Measurement error**			
Positive subscale	Generalizability coefficient range: 0.662–0.80 (total sample size: 319)	Indeterminate (4× (?))	Low (multiple inadequate studies available)
Negative subscale	Generalizability coefficient range: 0.647–0.77 (total sample size: 319)	Indeterminate (4× (?))	Low (multiple inadequate studies available)
General psychopathology subscale	Generalizability coefficient range: 0.691–0.84 (total sample size: 319)	Indeterminate (4× (?))	Low (multiple inadequate studies available)
PANSS-30 total score	Generalizability coefficient range: 0.687–0.93 (total sample size: 319)	Indeterminate (4× (?))	Low (multiple inadequate studies available)
**Criterion validity**			
PANSS-30 total score	Pooled: r = 0.877 (total sample size = 3415)	Sufficient	High
**Hypothesis testing**			
Positive subscale	36 out of 37 hypotheses confirmed	Sufficient	High
Negative subscale	37 out of 39 hypotheses confirmed	Sufficient	High
General psychopathology subscale	16 out of 20 hypotheses confirmed	Sufficient	High
PANSS-30 total score	5 out of 7 hypotheses confirmed	Sufficient	Moderate (due to inconsistent results)
**Responsiveness**			
PANSS-30 total score	6 out of 6 hypotheses confirmed	Sufficient	High

### Recommendations

The PANSS total is categorized as “not-recommendable” (C), the subscales fall in the “potential to be recommended” (B) category.

The PANSS total score is rated lower than the subscales due to strong evidence for “insufficient” structural validity. The subscales are not necessarily better, but there is less evidence available, which places them in the intermediate category “B”, as future evidence could hypothetically lead to a more positive evaluation. This is due to COSMIN considering each subscale as a scale of its own, requiring individual assessments. However, the categorization of the subscales in the intermediate category “B” needs be interpreted in the context of the lacking structural validity of the PANSS total score, which does not suggest a three-factor structure.

### Feasibility

Strengths of the PANSS are its availability in over 40 languages, simple score calculation by summing up item scores, the availability of the PANSS manual with its precise definitions, the Structured Clinical Interview for PANSS assessments (SCI-PANSS) and the Informant Questionnaire (IQ-PANSS).^141^, ^142^, ^143^ Weaknesses are the scale's long completion time of 30–50 min, requirements for rater training to achieve acceptable interrater reliability making the standardization process laborious, and the high cost of the instrument ($96,40 for the PANSS Technical Manual, $90,80 for 25 PANSS Rating and Profile Forms) due to copyright reasons.^1^^,^^114^^,^^144^ The European Medicines Agency (EMA) requires the use of the PANSS for drug approval (compare Appendix S8).^145^

## Discussion

This systematic COSMIN review and meta-analysis based on 118 publications showed that the PANSS is a strong scale in terms of reliability, construct validity, criterion validity, and responsiveness, which are important for treatment trials. Nevertheless, it also has major shortcomings. Its content validity is questionable because for the development of the PANSS, the BPRS was simply supplemented by items of the PRS rather than using a methodological scale construction process (e.g., derive the items statistically from a large item pool, involve clinicians and patients in the process, etc.). Structural validity is poor because the original 3 factor model has a bad statistical fit.

The satisfying responsiveness currently is a justification for the use of the PANSS in clinical trials and for the European Medicines Agency (EMA) as a requirement for drug approval.^145^ The ability to separate between drug and placebo (an aspect of responsiveness) is of key importance in such trials. Nevertheless, even very short scales such as the Clinical Global Impression Scale regularly separate between interventions.^146^ Other advantages of the PANSS are that its scores are relatively interpretable, although the linking analyses which made this possible were carried out late after its introduction.^119^^,^^125^^,^^126^^,^^147^, ^148^, ^149^, ^150^

Such analyses have shown that approximately 25% PANSS total score reduction from baseline means minimally improved according to the CGI, 50% much improved, and 15/5 absolute PANSS total/negative points reduction minimally improved according to the CGI.^119^^,^^125^^,^^148^, ^149^, ^150^ It should be noted that the 30 minimum points of the PANSS need to be subtracted when calculating percentage improvement because otherwise response is underestimated.^150^, ^151^, ^152^ Other strong points are the availability in more than 40 languages, a PANSS institute which among others provides training, and interview guides such as the SCI-PANSS, a structured clinical interview, and the IQ-PANSS, a structured informant questionnaire.^141^, ^142^, ^143^^,^^153^

One of the most frequently assessed and questioned aspects of the PANSS is its structural validity. Exploratory approaches extensively applied principal component analyses and exploratory factor analyses, but also more robust and more valid methods such as 10-fold-cross-validations, which clearly demonstrated the inaccuracy of the three-subscale structure.^42^^,^^59^ These analyses yielded a variety of models with factor numbers ranging between 4 and 9, but most analyses supported a five-factor-model solution. We will report our assessment of these factor models in a separate publication. We emphasize that the usefulness of the original negative subscale is particularly low. In the NIMH-MATRICS consensus, it was decided that negative symptoms encompass 5 domains (Blunted affect, Alogia, Asociality, Avolition, Anhedonia), of which the PANSS covers only the first three.^154^ Moreover, studies have shown that the negative subscale is not scalable and not unidimensional.^91^^,^^92^ In recent reviews, the modern scales for negative symptoms, Clinical Assessment Interview for Negative Symptoms (CAINS) and Brief negative Symptom Scale (BNSS), were overall better than older scales as the Scale for the Assessment of Negative Symptoms (SANS) and the Negative Symptom Assessment (NSA).^13^^,^^14^^,^^155^, ^156^, ^157^, ^158^, ^159^, ^160^ The PANSS negative subscale is also inferior to the CAINS and BNSS for the reasons just mentioned.

Due to unclear or inadequate structural validity, internal consistency is questionable as well. According to COSMIN good structural validity is a prerequisite for the interpretability of internal consistency statistics.^8^, ^9^, ^10^ High Cronbach's alpha values indicate a high interrelatedness of the items of the PANSS and its subscales, but if the structure of a scale is unclear the informative value is limited because the underlying structure may in reality be different. Good internal consistency is a prerequisite for a good (“A”) recommendation by COSMIN.^8^, ^9^, ^10^ Thus, if, for example, improved statistical models found a factor structure of the PANSS with better fit, the recommendability of the scale could increase.

These weaknesses have also implications on the interpretation of previous studies. In particular, as long as the content validity of a scale is not clear, we cannot be sure that we are asking the right questions. Having said this, the PANSS has not been designed as a diagnostic scale. Its main purpose is to be sensitive for change (“responsiveness”) and this is a strength of the PANSS. Nevertheless, PANSS total score-based effect sizes of antipsychotic drugs compared to placebo are relatively low (average ∼0.5; compare Leucht et al. (2017)) and might be higher with a better scale. In terms of structural validity, some studies have analyzed both the original 3 subscale structure and a 5-factor structure but did not find clear differences between them (e.g., Nemeth et al. (2017)).^161^^,^^162^ The PANSS does not include all domains found important by the NIMH consensus for negative symptoms. Thus, previous studies could not assess all these domains. In sum if a new scale were developed, it needs to be found out whether it would reach better results.

Finally, feasibility is problematic. An interview lasts about 45 min which precludes the use of the PANSS in routine clinical practice.^1^ The anchors for the ratings are clearly defined but as there are 30 items (i.e., 30 times 6 degrees = 180 definitions), only very experienced raters know them by heart.^142^ Moreover, it has been shown that not all items are scalable, i.e., not all items contribute additional information.^11^^,^^22^^,^^27^, ^28^, ^29^ This has led to the development of various short versions, such as the 14-item Mini-PANSS, the adapted 19-item Mini-PANSS, the PANSS-8, the six-item Brief PANSS, the PANSS-6,^11^^,^^29^^,^^49^^,^^55^^,^^163^, ^164^, ^165^ and the Brief Evaluation of Psychosis Symptom Domains (BE-PSD) scale.^166^ Five-factor solutions and PANSS-6 will be addressed in our companion reviews.^167^

This systematic review has several limitations.

First, while the COSMIN framework offers a structured approach to evaluating the measurement properties of instruments like the PANSS, it does not accommodate certain methodologies that might provide a more precise analysis. This required us to use certain workarounds to accommodate for the variety of approaches used and prevented us from assessing two studies that applied network analyses and orthonormal projective non-negative matrix factorization (OPNMF), both modern ways to assess scale structure.^108^^,^^168^ The first study found that the interconnection of symptoms appears to be different between treatment responsive versus treatment resistant patients.^168^ The second one supported a four-factor structure (see Appendix S9).^108^ Moreover, some old methods which have been frequently used are increasingly considered less appropriate (e.g., Cronbach's alpha for reliability).^169^

Thus, several methodologically different procedures with different levels of validity were assessed based on the same criteria, e.g., no consideration of different rotation methods or eigenvalue criteria, or different kinds of correlation coefficients entered calculations despite their differences.^170^ Nevertheless, such studies which could not be implemented in the COSMIN framework and were, therefore, rated as indeterminate “(?)”, yielded similar results. Only very few analyses from the 119 included studies could not be incorporated into the COSMIN framework, therefore their impact is minor. We recommend that in future versions of COSMIN new methods to assess rating scales will be implemented.

Another point is that the COSMIN framework mainly bases its final step “recommendation” on the domains “content validity” and “internal consistency”. Some may set this focus differently.

Second, the PANSS is an instrument with ordinal scaling, but as the original authors of the included studies we assumed that it is continuous. This problem applies to many psychiatric rating scales, but the implications are unclear.

Third, the populations, raters (degree of training) and the methods used in the studies were quite heterogeneous.

Fourth, we report a lack of evidence on responsiveness of the subscales in our review as none of the included validation studies assessed it. However, multiple regular studies applying the PANSS subscales could demonstrate its ability to detect pre- and post-treatment differences, but these would not have changed the overall rating of the PANSS.^162^^,^^171^

Fifth, our choice to consider the BPRS as gold standard for criterion validity might be questioned, as the PANSS was derived from the BPRS. If we had not considered the BPRS as the gold standard, the domain criterion validity would not have been assessable by COSMIN. Such a situation does not lead to a downgrading. Thus, the overall judgment would not have changed.

Sixth, although COSMIN is a highly elaborated and operationalized framework to systematically review the measurement properties of rating scales, there is a degree of subjectivity.^8^, ^9^, ^10^

The PANSS is a comprehensively evaluated tool with strengths in responsiveness, reliability and construct validity. The field also has vast experience with this scale. Nevertheless, there are important shortcomings primarily regarding content- and structural validity, which are the fundamental domains in the COSMIN approach. If only the strengths in terms of responsiveness and reliability of the PANSS are needed, it can be useful, but its weaknesses limit interpretation because content and structural validity determine the meaning of a score. Moreover, the length of the scale limits its use in daily clinical routine and in clinical trials. The original three-subscale structure is not valid. In addition to the short versions of the PANSS mentioned above, short scales such as the Brief Evaluation of Psychosis Symptom Domains (BE-PSD) and the Clinical Global Impression-Schizophrenia Scale may be more applicable in practice.^166^^,^^172^^,^^173^ Appropriately developed new scales for comprehensively and accurately assessing the symptoms of schizophrenia are warranted, possibly within a formative rather than reflective framework.

## Contributors

S.G. and M.R. contributed equally to this work and are both recognized as first authors. S.G. and M.R. had full access to all data in the study and take responsibility for verification of the data, and the accuracy of the data analysis. The authors confirm contribution to the paper as follows: study conception and design: S.G., M.R., and S.L.; data collection: S.G. and M.R.; analysis and interpretation of results: S.G., M.R., and S.L.; statistical consultation: M.B.; draft manuscript preparation: S.G. and M.R.; revision for important intellectual content: S.L., M.B., S.W., L.W., J.P., and J.M.D.; The work will be part of the doctoral thesis of S.G. and M.R.; All authors reviewed the results and approved the final version of the manuscript. All authors have agreed to be personally accountable for the author's own contributions and to ensure that questions related to the accuracy or integrity of any part of the work, are appropriately investigated, resolved, and the resolution documented in the literature.

## Data sharing statement

Underlying study data will be shared by the corresponding author (stefan.leucht@tum.de) on reasonable request for academic and research purposes and is subject to data sharing agreements. Supplementary data can be accessed in the Appendix.

## Declaration of generative AI and AI-assisted technologies in the writing process

During the preparation of this work the authors used ChatGPT-4o by OpenAI, Inc. to improve readability and comprehensibility. After using this tool, the authors reviewed and edited the content as needed and take full responsibility for the content of the publication.

## Declaration of interests

In the last 3 years, Dr Leucht has received honoraria for advising, consulting, and for lectures and/or for educational material from Angelini, Apsen, Boehringer Ingelheim, Eisai, Ekademia, GedeonRichter, Janssen, Karuna, Kynexis, Lundbeck, Medichem, Medscape, Mitsubishi, Neurotorium, Otsuka, Novo Nordisk, Recordati, Rovi, and Teva. All other authors declare that they have no conflicts of interest relevant to this research.

## References

[1] Kay S.R., Fiszbein A., Opler L.A. (1987). The positive and negative syndrome scale (PANSS) for schizophrenia. Schizophr Bull.

[2] Liechti S., Capodilupo G., Opler D.J., Opler M., Yang L.H. (2017). A developmental history of the positive and negative syndrome scale (PANSS). Innov Clin Neurosci.

[3] Crow T.J. (1980). Molecular pathology of schizophrenia: more than one disease process?. Br Med J.

[4] Crow T.J. (1980). Positive and negative schizophrenic symptoms and the role of dopamine. Br J Psychiatry.

[5] Mass R., Schoemig T., Hitschfeld K., Wall E., Haasen C. (2000). Psychopathological syndromes of schizophrenia: evaluation of the dimensional structure of the positive and negative syndrome scale. Schizophr Bull.

[6] Overall J.E., Gorham D.R. (1962). The brief psychiatric rating scale. Psychol Rep.

[7] Singh M.M., Kay S.R. (1975). A comparative study of haloperidol and chlorpromazine in terms of clinical effects and therapeutic reversal with benztropine in schizophrenia. Theoretical implications for potency differences among neuroleptics. Psychopharmacologia.

[8] Mokkink L.B., de Vet H.C.W., Prinsen C.A.C. (2018). COSMIN risk of bias checklist for systematic reviews of patient-reported outcome measures. Qual Life Res.

[9] Prinsen C.A.C., Mokkink L.B., Bouter L.M. (2018). COSMIN guideline for systematic reviews of patient-reported outcome measures. Qual Life Res.

[10] Terwee C.B., Prinsen C.A.C., Chiarotto A. (2018). COSMIN methodology for evaluating the content validity of patient-reported outcome measures: a Delphi study. Qual Life Res.

[11] Ostergaard S.D., Lemming O.M., Mors O., Correll C.U., Bech P. (2016). PANSS-6: a brief rating scale for the measurement of severity in schizophrenia. Acta Psychiatr Scand.

[12] Marder S.R., Davis J.M., Chouinard G. (1997). The effects of risperidone on the five dimensions of schizophrenia derived by factor analysis: combined results of the North American trials. J Clin Psychiatry.

[13] Weigel L., Wehr S., Galderisi S. (2023). The Brief negative Symptom Scale (BNSS): a systematic review of measurement properties. Schizophrenia (Heidelb).

[14] Wehr S., Weigel L., Davis J., Galderisi S., Mucci A., Leucht S. (2024). Clinical assessment interview for negative symptoms (CAINS): a systematic review of measurement properties. Schizophr Bull.

[15] Roithmeier M., Geck S., Peter N., Leucht S. (2024). Systematic review of the positive and negative syndrome scale following the COSMIN standard. https://osf.io/2t593.

[16] Terwee C.B., Jansma E.P., Riphagen I.I., de Vet H.C. (2009). Development of a methodological PubMed search filter for finding studies on measurement properties of measurement instruments. Qual Life Res.

[17] Hedlund J.L. (1980). The brief psychiatric rating scale (BPRS): a comprehensive review. J Oper Psychiatr.

[18] Elsman E.B.M., Mokkink L.B., Langendoen-Gort M. (2022). Systematic review on the measurement properties of diabetes-specific patient-reported outcome measures (PROMs) for measuring physical functioning in people with type 2 diabetes. BMJ Open Diabetes Res Care.

[19] Zúñiga Le-Bert M., Wiessner M., Wehr S., Weigel L., Leucht S. (2024). Schizophrenia quality of life scale and schizophrenia quality of life scale revision 4: a systematic review of measurement properties. Schizophr Bull.

[20] Borenstein M., Hedges L., Higgins J., Rothstein H. (2009).

[21] Findling R.L., Youngstrom E.A., McClellan J.M. (2023). An optimized version of the positive and negative symptoms scale (PANSS) for pediatric trials. J Am Acad Child Adolesc Psychiatry.

[22] Baandrup L., Allerup P., Nielsen M.O. (2022). Scalability of the Positive and Negative Syndrome Scale in first-episode schizophrenia assessed by Rasch models. Acta Psychiatr Scand.

[23] Lim K., Peh O.H., Yang Z. (2021). Large-scale evaluation of the positive and negative syndrome scale (PANSS) symptom architecture in schizophrenia. Asian J Psychiatr.

[24] Fountoulakis K.N., Dragioti E., Theofilidis A.T. (2019). Staging of schizophrenia with the use of PANSS: an international multi-center study. Int J Neuropsychopharmacol.

[25] Walsh-Messinger J., Antonius D., Opler M. (2018). Factor structure of the positive and negative syndrome scale (PANSS) differs by sex. Clin Schizophr Relat Psychoses.

[26] Petruzzelli M.G., Margari L., Bosco A., Craig F., Palumbi R., Margari F. (2018). Early onset first episode psychosis: dimensional structure of symptoms, clinical subtypes and related neurodevelopmental markers. Eur Child Adolesc Psychiatry.

[27] Ostergaard S.D., Foldager L., Mors O., Bech P., Correll C.U. (2018). The validity and sensitivity of PANSS-6 in treatment-resistant schizophrenia. Acta Psychiatr Scand.

[28] Ostergaard S.D., Foldager L., Mors O., Bech P., Correll C.U. (2018). The validity and sensitivity of PANSS-6 in the clinical antipsychotic trials of intervention effectiveness (CATIE) study. Schizophr Bull.

[29] Lin C.H., Lin H.S., Lin S.C., Kuo C.C., Wang F.C., Huang Y.H. (2018). Early improvement in PANSS-30, PANSS-8, and PANSS-6 scores predicts ultimate response and remission during acute treatment of schizophrenia. Acta Psychiatr Scand.

[30] Lefort-Besnard J., Varoquaux G., Derntl B. (2018). Patterns of schizophrenia symptoms: hidden structure in the PANSS questionnaire. Transl Psychiatry.

[31] Hopkins S.C., Ogirala A., Loebel A., Koblan K.S. (2018). Transformed PANSS factors intended to reduce pseudospecificity among symptom domains and enhance understanding of symptom change in antipsychotic-treated patients with schizophrenia. Schizophr Bull.

[32] Grover S., Dua D., Chakrabarti S., Avasthi A. (2018). Factor analysis of symptom dimensions (psychotic, affective and obsessive compulsive symptoms) in schizophrenia. Asian J Psychiatr.

[33] De Freitas R., Dos Santos B., Altamura C. (2018). Can the positive and negative syndrome scale (PANSS) differentiate refractory from non-refractory schizophrenia? A factor analytic investigation based on data from the pattern cohort study. Schizophr Bull.

[34] Anderson A.E., Marder S., Reise S.P. (2018). Bifactor modeling of the positive and negative syndrome scale: generalized psychosis spans schizoaffective, bipolar, and schizophrenia diagnoses. Schizophr Bull.

[35] Yehya A., Ghuloum S., Mahfoud Z. (2017). Validation of the five-factor model of the Arabic version of the positive and negative syndrome scale in schizophrenia. Psychopathology.

[36] Peitl V., Štefanović M., Karlović D. (2017). Depressive symptoms in schizophrenia and dopamine and serotonin gene polymorphisms. Prog Neuropsychopharmacol Biol Psychiatry.

[37] Dragioti E., Wiklund T., Siamouli M., Moutou K., Fountoulakis K.N. (2017). Could PANSS be a useful tool in the determining of the stages of schizophrenia? A clinically operational approach. J Psychiatr Res.

[38] Anderson A.E., Reise S.P., Marder S.R., Mansolf M., Han C., Bilder R.M. (2017). Disparity between general symptom relief and remission criteria in the positive and negative syndrome scale (PANSS): a post-treatment bifactor item response theory model. Innov Clin Neurosci.

[39] Thokagevistk K., Millier A., Lenert L., Sadikhov S., Moreno S., Toumi M. (2016). Validation of disease states in schizophrenia: comparison of cluster analysis between US and European populations. J Market Access Health Policy.

[40] Best M.W., Grossman M., Oyewumi L.K., Bowie C.R. (2016). Examination of the Positive and Negative Syndrome Scale factor structure and longitudinal relationships with functioning in early psychosis. Early Interv Psychiatry.

[41] Xu K., Krystal J.H., Ning Y. (2015). Preliminary analysis of positive and negative syndrome scale in ketamine-associated psychosis in comparison with schizophrenia. J Psychiatr Res.

[42] Anderson A., Wilcox M., Savitz A. (2015). Sparse factors for the positive and negative syndrome scale: which symptoms and stage of illness?. Psychiatry Res.

[43] Stochl J., Jones P.B., Plaistow J. (2014). Multilevel ordinal factor analysis of the positive and negative syndrome scale (PANSS). Int J Methods Psychiatr Res.

[44] Higuchi C.H., Ortiz B., Berberian A.A. (2014). Factor structure of the positive and negative syndrome scale (PANSS) in Brazil: convergent validation of the Brazilian version. Br J Psychiatry.

[45] Jiang J., Sim K., Lee J. (2013). Validated five-factor model of positive and negative syndrome scale for schizophrenia in Chinese population. Schizophr Res.

[46] Kumar A., Khess C.R.J. (2012). Factor analysis of positive and negative syndrome scale in schizophrenia: an exploratory study. Indian J Psychiatry.

[47] Kim J.H., Kim S.Y., Lee J., Oh K.J., Kim Y.B., Cho Z.H. (2012). Evaluation of the factor structure of symptoms in patients with schizophrenia. Psychiatry Res.

[48] Levine S.Z., Rabinowitz J., Rizopoulos D. (2011). Recommendations to improve the positive and negative syndrome scale (PANSS) based on item response theory. Psychiatry Res.

[49] Khan A., Lewis C., Lindenmayer J.P. (2011). Use of non-parametric item response theory to develop a shortened version of the Positive and Negative Syndrome Scale (PANSS). BMC Psychiatry.

[50] Citrome L., Meng X., Hochfeld M. (2011). Efficacy of iloperidone in schizophrenia: a PANSS five-factor analysis. Schizophr Res.

[51] Hwang S.S.H., Chang J.S., Lee K.Y., Ahn Y.M., Kim Y.S. (2009). The causal model of insight in schizophrenia based on the positive and negative syndrome scale factors and the structural equation modeling. J Nerv Ment Dis.

[52] Gil D., Bengochea R., Arrieta M. (2009). Validity of the cognitive factor of the Positive and Negative Syndrome Scale as a measure of cognitive functioning in schizophrenia. Rev Psiquiatr Salud Ment.

[53] Ruiz-Veguilla M., Cervilla J.A., Barrigon M.L. (2008). Neurodevelopmental markers in different psychopathological dimensions of first episode psychosis: the ESPIGAS study. Eur Psychiatry.

[54] Lindenmayer J.P., Bossie C.A., Kujawa M., Zhu Y., Canuso C.M. (2008). Dimensions of psychosis in patients with bipolar mania as measured by the positive and negative syndrome scale. Psychopathology.

[55] Santor D.A., Ascher-Svanum H., Lindenmayer J.P., Obenchain R.L. (2007). Item response analysis of the positive and negative syndrome scale. BMC Psychiatry.

[56] Ruhrmann S., Kissling W., Lesch O.M., Schmauss M., Seemann U., Philipp M. (2007). Efficacy of flupentixol and risperidone in chronic schizophrenia with predominantly negative symptoms. Prog Neuropsychopharmacol Biol Psychiatry.

[57] Levine S.Z., Rabinowitz J. (2007). Revisiting the 5 dimensions of the positive and negative syndrome scale. J Clin Psychopharmacol.

[58] Villalta-Gil V., Vilaplana M., Ochoa S. (2006). Four symptom dimensions in outpatients with schizophrenia. Compr Psychiatry.

[59] van der Gaag M., Hoffman T., Remijsen M. (2006). The five-factor model of the Positive and Negative Syndrome Scale II: a ten-fold cross-validation of a revised model. Schizophr Res.

[60] Tirupati S.N., Padmavati R., Thara R., McCreadie R.G. (2006). Psychopathology in never-treated schizophrenia. Compr Psychiatry.

[61] Reichenberg A., Rieckmann N., Harvey P.D. (2005). Stability in schizophrenia symptoms over time: findings from the Mount Sinai Pilgrim Psychiatric Center Longitudinal Study. J Abnorm Psychol.

[62] Fresán A., De la Fuente-Sandoval C., Loyzaga C. (2005). A forced five-dimensional factor analysis and concurrent validity of the Positive and Negative Syndrome Scale in Mexican schizophrenic patients. Schizophr Res.

[63] Mohr P.E., Cheng C.M., Claxton K. (2004). The heterogeneity of schizophrenia in disease states. Schizophr Res.

[64] Masiak M., Loza B. (2004). Core factors of schizophrenia structure based on PANSS and SAPS/SANS results. Discerning and head-to-head comparison of PANSS and SASPS/SANS validity. Psychiatr Pol.

[65] Lindenmayer J.P., Czobor P., Volavka J. (2004). Effects of atypical antipsychotics on the syndromal profile in treatment-resistant schizophrenia. J Clin Psychiatry.

[66] Lee K.H., Harris A.W., Loughland C.M., Williams L.M. (2003). The five symptom dimensions and depression in schizophrenia. Psychopathology.

[67] Honey G.D., Sharma T., Suckling J. (2003). The functional neuroanatomy of schizophrenic subsyndromes. Psychol Med.

[68] Emsley R., Rabinowitz J., Torreman M. (2003). The factor structure for the Positive and Negative Syndrome Scale (PANSS) in recent-onset psychosis. Schizophr Res.

[69] Drake R.J., Dunn G., Tarrier N., Haddock G., Haley C., Lewis S. (2003). The evolution of symptoms in the early course of non-affective psychosis. Schizophr Res.

[70] El Yazaji M., Battas O., Agoub M. (2002). Validity of the depressive dimension extracted from principal component analysis of the PANSS in drug-free patients with schizophrenia. Schizophr Res.

[71] Wolthaus J.E., Dingemans P.M., Schene A.H. (2000). Component structure of the Positive and Negative Syndrome Scale (PANSS) in patients with recent-onset schizophrenia and spectrum disorders. Psychopharmacology (Berl).

[72] Lykouras L., Oulis P., Psarros K. (2000). Five-factor model of schizophrenic psychopathology: how valid is it?. Eur Arch Psychiatry Clin Neurosci.

[73] Lançon C., Auquier P., Nayt G., Reine G. (2000). Stability of the five-factor structure of the positive and negative syndrome scale (PANSS). Schizophr Res.

[74] Lançon C., Reine G., Llorca P.M., Auquier P. (1999). Validity and reliability of the French-language version of the positive and negative syndrome scale (PANSS). Acta Psychiatr Scand.

[75] Bunk D., Eggers C., Klapal M. (1999). Symptom dimensions in the course of childhood-onset schizophrenia. Eur Child Adolesc Psychiatry.

[76] Lançon C., Aghababian V., Llorca P.M., Auquier P. (1998). Factorial structure of the Positive and Negative Syndrome Scale (PANSS): a forced five-dimensional factor analysis. Acta Psychiatr Scand.

[77] Klapal M., Eggers C., Bunk D., Koriath H. (1998). [The 5 factor model of childhood schizophrenia]. Nervenarzt.

[78] Higashima M., Urata K., Kawasaki Y. (1998). P300 and the thought disorder factor extracted by factor-analytic procedures in schizophrenia. Biol Psychiatry.

[79] Loas G., Noisette C., Legrand A., Delahousse J. (1997). A four-syndrome model of chronic schizophrenia: principal components analysis of the Positive and Negative Syndrome Scale (PANSS) in 153 chronic schizophrenics. Encephale.

[80] Lancon C., Auquier P., Llorca P.M., Martinez J.L., Bougerol T., Scotto J.C. (1997). Psychometric properties of the PANSS in a sample of French schizophrenic patients. Encephale.

[81] Dollfus S., Petit M. (1995). Principal-component analyses of PANSS and SANS-SAPS in schizophrenia: their stability in an acute phase. Eur Psychiatry.

[82] Peralta V. (1994). Psychometric properties of the positive and negative syndrome scale (PANSS) in schizophrenia. Psychiatry Res.

[83] Lindenmayer J.P., Bernstein-Hyman R., Grochowski S. (1994). A new five factor model of schizophrenia. Psychiatr Q.

[84] Lindenmayer J.P., Bernstein-Hyman R., Grochowski S. (1994). Five-factor model of schizophrenia. Initial validation. J Nerv Ment Dis.

[85] Kawasaki Y., Maeda Y., Sakai N. (1994). Evaluation and interpretation of symptom structures in patients with schizophrenia. Acta Psychiatr Scand.

[86] Bell M.D. (1994). Five-component model of schizophrenia: assessing the factorial invariance of the positive and negative syndrome scale. Psychiatry Res.

[87] Lindström E., von Knorring L. (1993). Principal component analysis of the Swedish version of the positive and negative syndrome scale for schizophrenia. Nord J Psychiatr.

[88] Lepine J. (1991). Dimensions positives et négatives dans les schizophrénies. Les Cahiers de Prisme.

[89] Dollfus S., Petit M., Lesieur P., Menard J.F. (1991). Principal-component analysis of PANSS and SANS-SAPS global ratings in schizophrenic patients. Eur Psychiatry.

[90] Kay S.R., Sevy S. (1990). Pyramidical model of schizophrenia. Schizophr Bull.

[91] Baandrup L., Allerup P., Nielsen M.O. (2020). Rasch analysis of the PANSS negative subscale and exploration of negative symptom trajectories in first-episode schizophrenia - data from the OPTiMiSE trial. Psychiatry Res.

[92] Cruz B.F., de Resende C.B., Abreu M.N. (2013). How specific are negative symptoms and cognitive impairment in schizophrenia? An analysis of PANSS and SCoRS. Cogn Neuropsychiatry.

[93] Lindstrom E., Wieselgren I.M., von Knorring L. (1994). Interrater reliability of the structured clinical interview for the positive and negative syndrome scale for schizophrenia. Acta Psychiatr Scand.

[94] Barrio C., Yamada A.M., Atuel H. (2003). A tri-ethnic examination of symptom expression on the positive and negative syndrome scale in schizophrenia spectrum disorders. Schizophr Res.

[95] Ivanova E., Khan A., Liharska L. (2018). Validation of the Russian version of the positive and negative syndrome scale (PANSS-Ru) and normative data. Innov Clin Neurosci.

[96] Hashimoto N., Takahashi K., Fujisawa D. (2020). A pilot validation study of the Japanese translation of the Positive and Negative Syndrome Scale (PANSS). Asian J Psychiatr.

[97] Li L., Ma H., Wang X., Meng E. (2021). Validation of Chinese version of positive and negative syndrome scale-6 in clinical setting: a preliminary study. Psychiatry Clin Psychopharmacol.

[98] Medvedev O.N., Berk M., Dean O.M. (2021). A novel way to quantify schizophrenia symptoms in clinical trials. Eur J Clin Invest.

[99] Kay S.R., Fiszbein A., Lindenmayer J.P., Opler L.A. (1986). Positive and negative syndromes in schizophrenia as a function of chronicity. Acta Psychiatr Scand.

[100] Kay S.R., Opler L.A., Fiszbein A. (1986). Significance of positive and negative syndromes in chronic schizophrenia. Br J Psychiatry.

[101] Bell M., Milstein R., Beam-Goulet J., Lysaker P., Cicchetti D. (1992). The positive and negative syndrome scale and the brief psychiatric rating scale. Reliability, comparability, and predictive validity. J Nerv Ment Dis.

[102] von Knorring L., Lindström E. (1992). The Swedish version of the Positive and Negative Syndrome Scale (PANSS) for schizophrenia. Construct validity and interrater reliability. Acta Psychiatr Scand.

[103] Peralta V., Cuesta M.J., de Leon J. (1995). Positive and negative symptoms/syndromes in schizophrenia: reliability and validity of different diagnostic systems. Psychol Med.

[104] Vollema M.G., Geurtsen G.J., Kuipers T. (1995). Negative symptoms: a unidimensional construct related to frontal lobe deficits. Tijdschr Psychiatr.

[105] Norman R.M., Malla A.K., Cortese L., Diaz F. (1996). A study of the interrelationship between and comparative interrater reliability of the SAPS, SANS and PANSS. Schizophr Res.

[106] Igarashi Y., Hayashi N., Yamashina M. (1998). Interrater reliability of the Japanese version of the Positive and Negative Syndrome Scale and the appraisal of its training effect. Psychiatry Clin Neurosci.

[107] Anıl Yağcıoğlu A.E., Batur S., Tiryaki A., Gogus A. (1999). Reliability and validity of the Turkish version of the positive and negative syndrome scale (PANSS). Türk Psikol Derg.

[108] Chen J., Patil K.R., Weis S. (2020). Neurobiological divergence of the positive and negative schizophrenia subtypes identified on a new factor structure of psychopathology using non-negative factorization: an international machine learning study. Biol Psychiatry.

[109] Kay S.R., Fiszbein A., Vital-Herne M., Fuentes L.S. (1990). The positive and negative syndrome scale - Spanish adaptation. J Nerv Ment Dis.

[110] Nilchaikovit T., Uneanong S., Kessawai D., Thomyangkoon P. (2000). The Thai version of the Positive and Negative Syndrome Scale (PANSS) for schizophrenia: criterion validity and interrater reliability. J Med Assoc Thai.

[111] Aggarwal N.K., Tao H., Xu K., Stefanovics E., Zhening L., Rosenheck R.A. (2011). Comparing the PANSS in Chinese and American inpatients: cross-cultural psychiatric analyses of instrument translation and implementation. Schizophr Res.

[112] Aggarwal N.K., Zhang X.Y., Stefanovics E. (2012). Rater evaluations for psychiatric instruments and cultural differences: the positive and negative syndrome scale in China and the United States. J Nerv Ment Dis.

[113] Bentsen H., Munkvold O.G., Notland T.H. (1996). The interrater reliability of the positive and negative syndrome scale (PANSS). Int J Methods Psychiatr Res.

[114] Müller M.J., Rossbach W., Dannigkeit P., Müller-Siecheneder F., Szegedi A., Wetzel H. (1998). Evaluation of standardized rater training for the positive and negative syndrome scale (PANSS). Schizophr Res.

[115] Müller M.J., Wetzel H. (1998). Improvement of inter-rater reliability of PANSS items and subscales by a standardized rater training. Acta Psychiatr Scand.

[116] Khan A., Yavorsky W.C., Liechti S. (2013). Assessing the sources of unreliability (rater, subject, time-point) in a failed clinical trial using items of the Positive and Negative Syndrome Scale (PANSS). J Clin Psychopharmacol.

[117] Kay S.R., Opler L.A., Lindenmayer J.P. (1988). Reliability and validity of the positive and negative syndrome scale for schizophrenics. Psychiatry Res.

[118] Jelastopulu E., Giourou E., Merekoulias G., Mestousi A., Moratis E., Alexopoulos E.E.C. (2013). Correlation between the personal and social performance scale (PSP) and the positive and negative syndrome scale (PANSS) in a Greek sample of patients with schizophrenia. BMC Psychiatry.

[119] Leucht S., Kane J.M., Kissling W., Hamann J., Etschel E., Engel R.R. (2005). What does the PANSS mean?. Schizophr Res.

[120] Edgar C.J., Blaettler T., Bugarski-Kirola D., Le Scouiller S., Garibaldi G.M., Marder S.R. (2014). Reliability, validity and ability to detect change of the PANSS negative symptom factor score in outpatients with schizophrenia on select antipsychotics and with prominent negative or disorganized thought symptoms. Psychiatry Res.

[121] Callegari C., Poloni N., Pace V. (2005). Evaluation of positive and negative symptoms in chronic schizophrenia: an interrelationship study among SANS/SAPS, PANSS and InSka rating scales. Minerva Psichiatr.

[122] Wallwork R.S., Fortgang R., Hashimoto R., Weinberger D.R., Dickinson D. (2012). Searching for a consensus five-factor model of the positive and negative syndrome scale for schizophrenia. Schizophr Res.

[123] Van Erp T.G., Preda A., Nguyen D. (2014). Converting positive and negative symptom scores between PANSS and SAPS/SANS. Schizophr Res.

[124] Hieronymus F., Correll C.U., Østergaard S.D. (2023). Initial severity of the Positive and Negative Syndrome Scale (PANSS)-30, its main subscales plus the PANSS-6, and the relationship to subsequent improvement and trial dropout: a pooled participant-level analysis of 18 placebo-controlled risperidone and paliperidone trials. Transl Psychiatry.

[125] Leucht S., Kane J.M., Etschel E., Kissling W., Hamann J., Engel R.R. (2006). Linking the PANSS, BPRS, and CGI: clinical implications. Neuropsychopharmacology.

[126] Leucht S., Rothe P., Davis J.M., Engel R.R. (2013). Equipercentile linking of the BPRS and the PANSS. Eur Neuropsychopharmacol.

[127] Hieronymus F., Kølbæk P., Correll C.U., Østergaard S.D. (2021). Antipsychotic-placebo separation on the PANSS-6 subscale as compared to the PANSS-30: a pooled participant-level analysis. NPJ Schizophr.

[128] White L., Harvey P.D., Opler L., Lindenmayer J.P. (1997). Empirical assessment of the factorial structure of clinical symptoms in schizophrenia. A multisite, multimodel evaluation of the factorial structure of the Positive and Negative Syndrome Scale. The PANSS Study Group. Psychopathology.

[129] Fitzgerald P.B., de Castella A.R., Brewer K. (2003). A confirmatory factor analytic evaluation of the pentagonal PANSS model. Schizophr Res.

[130] Langeveld J., Andreassen O.A., Auestad B. (2013). Is there an optimal factor structure of the Positive and Negative Syndrome Scale in patients with first-episode psychosis?. Scand J Psychol.

[131] Rodriguez-Jimenez R., Bagney A., Mezquita L. (2013). Cognition and the five-factor model of the positive and negative syndrome scale in schizophrenia. Schizophr Res.

[132] Stefanovics E.A., Elkis H., Zhening L., Zhang X.Y., Rosenheck R.A. (2014). A cross-national factor analytic comparison of three models of PANSS symptoms in schizophrenia. Psychiatry Res.

[133] Woodward T.S., Jung K., Smith G.N. (2014). Symptom changes in five dimensions of the Positive and Negative Syndrome Scale in refractory psychosis. Eur Arch Psychiatr Clin Neurosci.

[134] Fong T.C., Ho R.T., Wan A.H., Siu P.J., Au-Yeung F.S. (2015). Psychometric validation of the consensus five-factor model of the positive and negative syndrome scale. Compr Psychiatry.

[135] Bentler P.M. (1990). Comparative fit indexes in structural models. Psychol Bull.

[136] Taber K.S. (2018). The use of Cronbach's alpha when developing and reporting research instruments in science education. Res Sci Educ.

[137] Cronbach L.J. (1951). Coefficient alpha and the internal structure of tests. Psychometrika.

[138] Ratner B. (2009). The correlation coefficient: its values range between +1/−1, or do they?. J Target Meas Anal Market.

[139] O'Brien R.M. (1995). Generalizability coefficients are reliability coefficients. Qual Quantity.

[140] Bloch R., Norman G. (2012). Generalizability theory for the perplexed: a practical introduction and guide: AMEE Guide No. 68. Med Teach.

[141] Kay S.R., Opler L.A., Lindenmayer J. (1992).

[142] Kay S.R., Opler L.A., Fiszbein A. (2006).

[143] Khan A., Liharska L., Harvey P.D., Atkins A., Ulshen D., Keefe R.S.E. (2017). Negative symptom dimensions of the positive and negative syndrome scale across geographical regions: implications for social, linguistic, and cultural consistency. Innov Clin Neurosci.

[144] Pearson Assessments (2023). Positive and negative syndrome scale. https://www.pearsonassessments.com/store/usassessments/en/Store/Professional-Assessments/Personality-%26-Biopsychosocial/Positive-and-Negative-Syndrome-Scale/p/P100025000.html.

[145] European Medicines Agency (2012).

[146] Leucht S., Engel R.R. (2006). The relative sensitivity of the clinical global impressions scale and the brief psychiatric rating scale in antipsychotic drug trials. Neuropsychopharmacology.

[147] Siafis S., Brandt L., McCutcheon R.A. (2024). Relapse in clinically stable adult patients with schizophrenia or schizoaffective disorder: evidence-based criteria derived by equipercentile linking and diagnostic test accuracy meta-analysis. Lancet Psychiatry.

[148] Leucht S., Barabássy Á., Laszlovszky I. (2019). Linking PANSS negative symptom scores with the Clinical Global Impressions Scale: understanding negative symptom scores in schizophrenia. Neuropsychopharmacology.

[149] Levine S.Z., Rabinowitz J., Engel R., Etschel E., Leucht S. (2008). Extrapolation between measures of symptom severity and change: an examination of the PANSS and CGI. Schizophr Res.

[150] Leucht S., Davis J.M., Engel R.R., Kane J.M., Wagenpfeil S. (2007). Defining ‘response' in antipsychotic drug trials: recommendations for the use of scale-derived cutoffs. Neuropsychopharmacology.

[151] Leucht S., Davis J.M., Engel R.R., Kissling W., Kane J.M. (2009). Definitions of response and remission in schizophrenia: recommendations for their use and their presentation. Acta Psychiatr Scand Suppl.

[152] Obermeier M., Mayr A., Schennach-Wolff R., Seemüller F., Möller H.J., Riedel M. (2010). Should the PANSS be rescaled?. Schizophr Bull.

[153] The PANSS Institute (2024). PANSS.org. https://panss.org/services-training.php.

[154] Marder S.R., Kirkpatrick B. (2014). Defining and measuring negative symptoms of schizophrenia in clinical trials. Eur Neuropsychopharmacol.

[155] Kring A.M., Gur R.E., Blanchard J.J., Horan W.P., Reise S.P. (2013). The clinical assessment interview for negative symptoms (CAINS): final development and validation. Am J Psychiatry.

[156] Kirkpatrick B., Strauss G.P., Nguyen L. (2011). The brief negative symptom scale: psychometric properties. Schizophr Bull.

[157] Horan W.P., Kring A.M., Gur R.E., Reise S.P., Blanchard J.J. (2011). Development and psychometric validation of the clinical assessment interview for negative symptoms (CAINS). Schizophr Res.

[158] Forbes C., Blanchard J.J., Bennett M., Horan W.P., Kring A., Gur R. (2010). Initial development and preliminary validation of a new negative symptom measure: the Clinical Assessment Interview for Negative Symptoms (CAINS). Schizophr Res.

[159] Alphs L.D., Summerfelt A., Lann H., Muller R.J. (1989). The negative symptom assessment: a new instrument to assess negative symptoms of schizophrenia. Psychopharmacol Bull.

[160] Andreasen N.C. (1983).

[161] Németh G., Laszlovszky I., Czobor P. (2017). Cariprazine versus risperidone monotherapy for treatment of predominant negative symptoms in patients with schizophrenia: a randomised, double-blind, controlled trial. Lancet.

[162] Leucht S., Leucht C., Huhn M. (2017). Sixty years of placebo-controlled antipsychotic drug trials in acute schizophrenia: systematic review, Bayesian meta-analysis, and meta-regression of efficacy predictors. Am J Psychiatry.

[163] Lindenmayer J.P. (2017). Are shorter versions of the positive and negative syndrome scale (PANSS) doable? A critical review. Innov Clin Neurosci.

[164] Yamamoto N., Inada T., Shimodera S., Morokuma I., Furukawa T.A. (2010). Brief PANSS to assess and monitor the overall severity of schizophrenia. Psychiatr Clin Neurosci.

[165] Andreasen N.C., William T., Carpenter J. (2005). Remission in schizophrenia: proposed criteria and rationale for consensus. Am J Psychiatry.

[166] Takeuchi H., Lee J., Fervaha G., Agid O., Remington G. (2023). Second version of brief evaluation of psychosis symptom domains (BE-PSD-V2.0). Schizophr Res.

[167] Geck S., Roithmeier M., Bühner M. (2025). COSMIN systematic review and meta-analysis of the measurement properties of the PANSS-6. Eur Neuropsychopharmacol.

[168] Esfahlani F.Z., Sayama H., Visser K.F., Strauss G.P. (2017). Sensitivity of the positive and negative syndrome scale (PANSS) in detecting treatment effects via network analysis. Innov Clin Neurosci.

[169] Kalkbrenner M.T. (2023). Alpha, omega, and H internal consistency reliability estimates: reviewing these options and when to use them. Counsel Outcome Res Eval.

[170] Fabrigar L.R., Wegener D.T., Maccallum R., Strahan E.J. (1999). Evaluating the use of exploratory factor analysis in psychological research. Psychol Methods.

[171] Huhn M., Nikolakopoulou A., Schneider-Thoma J. (2019). Comparative efficacy and tolerability of 32 oral antipsychotics for the acute treatment of adults with multi-episode schizophrenia: a systematic review and network meta-analysis. Lancet.

[172] Takeuchi H., Fervaha G., Lee J., Agid O., Remington G. (2016). A preliminary examination of the validity and reliability of a new brief rating scale for symptom domains of psychosis: brief Evaluation of Psychosis Symptom Domains (BE-PSD). J Psychiatr Res.

[173] Haro J.M., Kamath S.A., Ochoa S. (2003). The Clinical Global Impression-Schizophrenia scale: a simple instrument to measure the diversity of symptoms present in schizophrenia. Acta Psychiatr Scand Suppl.

