# Supplementary material for: COSMIN systematic review and meta-analysis of the measurement properties of the Positive and Negative Syndrome Scale (PANSS)
Source: eClinicalMedicine. 2025 Apr 11;82:103155. doi: 10.1016/j.eclinm.2025.103155 (PMC12008685; doi:10.1016/j.eclinm.2025.103155)
Supplement: Appendix S1 [file mmc1.docx]

**Appendix 1. OSF Study Protocol**

**Systematic review of the Positive and Negative Syndrome Scale following the COSMIN standard**

By Simon Geck, Maximilian Roithmeier, Stefan Leucht

Publication date: 2024-01-31

Publisher: Center for Open Science

*Review question:* How suitable are the Positive and Negative Syndrome Scale (PANSS), its Positive and Negative Subscores and short forms / patient-versions to evaluate/capture/document the symptom severity in patients with schizophrenia? 

*Search sources:* MEDLINE via PubMED, EMBASE. Additionally, screening of the reference lists of included studies for further publications. No other sources including unpublished studies will be sought. No restrictions on publication period. Articles will be included independent of the language they are in. According to the COSMIN standard only full text articles will be included.

*Search dates:* from database inception until 09.10.2023.

*Types of study to be included:* Any study that reports on the measurement properties of PANSS- Versions ( i.e. PANSS-total, PANSS-neg, PANSS-patient, PANSS-6) examined by us. Only published full-text peer-reviewed articles or manuals will be included.
Condition or domain being studied Symptom severity in terms of symptoms of schizophrenia in patients with schizophrenia spectrum disorders.

*Participants/population:* No restriction on age or phase of disease.

*Assessed instruments:* Clinician- or patient-reported rating scales that assess disease-related symptom severity, such as PANSS-total, PANSS-6, PANSS-patients. 

*Context:* Studies that report on the development and/or psychometric evaluation of observer-reported/ self-reported measures that assess disease-specific severity of symptoms will be included. No restrictions regarding setting.


**Procedure:**
Following the COSMIN manual, the following steps described in said manual will be conducted by two reviewers (SG and MR respectively) independently:

**Risk of bias assessment:**
The methodological quality of individual studies will be assessed using the COSMIN Risk of Bias checklist (Mokkink et al., 2018) consisting of 10 boxes which consist of several standards. The checklist will be conducted for each publication separately. Only the corresponding boxes of psychometric properties evaluated by the respective publication will be evaluated. The following boxes are included in the COSMIN Risk of Bias checklist:
- Box 1. Standards for evaluating the quality of PROM development
- Box 2. Standards for evaluating the quality of content validity studies of PROMs
- Box 3. Structural validity
- Box 4. Internal consistency
- Box 5. Cross-cultural validity/ measurement invariance
- Box 6. Reliability
- Box 7. Measurement error
- Box 8. Criterion validity
- Box 9. Hypotheses testing for construct validity
- Box 10. Responsiveness
After independently conducting the ratings, both reviewers will try to find consensus on their ratings. If consensus cannot be reached, a third, professor level reviewer (SL) will be consulted.

**Criteria for good measurement properties:**
Following the Risk of bias assessment, the characteristics of the included study population as well as the characteristics of the included PROMs will be summarized in two tables. For each of the included references the characteristics of the included validation studies will be described as exemplary shown in Appendix 3 respectively Appendix 4 of the COSMIN manual, , i.e. construct(s), target population, mode of administration, case numbers, gender distribution, ae mean (SD, range), disease severity etc..
Afterwards the result of each study on a measurement property will be rated against the updated criteria for good measurement properties (Terwee et al., 2007). Hereby each result is to be evaluated as either sufficient (+), insufficient (-), inconsistent (#), or indeterminate (?) (Prinsen et al., 2018).

**Summarization of evidence and grading of the evidences quality:**
While the Risk of bias assessment and the updated criteria for good measurement properties focus on the quality of individual studies, afterwards the quality of the whole PROM/ ClinROM will be assessed. Therefor the evidence will be summarized by, if the data is consistent, quantitatively pooling the results. The pooled results will then, as described above for individual studies, be compared to the updated criteria for good measurement properties. The quality of the resulting evidence will then be graded as either high, moderate, low or very low according to a modified GRADE (Grades of Recommendation, Assessment, Development and Evaluation) approach (see GRADE Handbook, 2013).

**Recommendations:**
If more instruments would be included in a COSMIN review, recommendations on the most suitable one for use in evaluative application would be formulated. As we just assess the PANSS scale, said scale will just be assigned to a category from A to C. PROMS(/ClinROMS) in category 'A' can be recommended for use and their results can be trusted. PROMs(/ClinROMs) categorized as ‘B’ have potential to be recommended for use, but further research is necessary to assess the quality of these instruments. PROMS(/ClinROMS) in category 'C' can't be recommended for use.

As last step, our systematic review depicting our findings will be reported.
