# Supplementary material for: COSMIN systematic review and meta-analysis of the measurement properties of the Positive and Negative Syndrome Scale (PANSS)
Source: eClinicalMedicine. 2025 Apr 11;82:103155. doi: 10.1016/j.eclinm.2025.103155 (PMC12008685; doi:10.1016/j.eclinm.2025.103155)
Supplement: Appendix S2 [file mmc2.docx]

**Appendix 2.** Search filters for EMBASE and MEDLINE

EMBASE

1 "Positive and Negative Syndrome Scale"/ or (PANSS* or "Positive and Negative Symptom" or "Positive and Negative Symptoms" or "Positive and Negative Syndrome" or "Positive and Negative Syndromes").mp. (21483)

2 exp Schizophrenia Spectrum Disorder/ or exp Schizophrenia/ or Schiz*.mp. (277621)

4 exp intermethod comparison/ or exp data collection method/ or exp validation study/ or exp feasibility study/ or exp pilot study/ or exp psychometry/ or exp reproducibility/ or exp observer variation/ or exp discriminant analysis/ or exp validity/ or (reproducib* or audit or psychometr* or clinimetr* or clinometr* or observer variation or reliab* or valid* or coefficient or internal consistency or (cronbach* and (alpha or alphas)) or item correlation or item correlations or item selection or item selections or item reduction or item reductions or agreement or precision or imprecision or precise values or test-retest or (test and retest) or (reliab* and (test or retest)) or stability or interrater or inter-rater or intrarater or intra-rater or intertester or inter-tester or intratester or intratester or interobeserver or inter-observer or intraobserver or intraobserver or intertechnician or inter-technician or intratechnician or intratechnician or interexaminer or inter-examiner or intraexaminer or intraexaminer or interassay or inter-assay or intraassay or intra-assay or interindividual or inter-individual or intraindividual or intra-individual or interparticipant or inter-participant or intraparticipant or intraparticipant or kappa or kappas or coefficient of variation or repeatab* or ((replicab* or repeated) and (measure or measures or findings or result or results or test or tests)) or generaliza* or generalisa* or concordance or (intraclass and correlation*) or discriminative or known group or factor analysis or factor analyses or factor structure or factor structures or dimensionality or subscale* or multitrait scaling analysis or multitrait scaling analyses or item discriminant or interscale correlation or interscale correlations or ((error or errors) and (measure* or correlat* or evaluat* or accuracy or accurate or precision or mean)) or individual variability or interval variability or rate variability or variability analysis or (uncertainty and (measurement or measuring)) or standard error of measurement or sensitiv* or responsive* or (limit and detection) or minimal detectable concentration or interpretab* or (small* and (real or detectable) and (change or difference)) or meaningful change or minimal important change or minimal important difference or minimally important change or minimally important difference or minimal detectable change or minimal detectable difference or minimally detectable change or minimally detectable difference or minimal real change or minimal real difference or minimally real change or minimally real difference or ceiling effect or floor effect or item response model or irt or rasch or differential item functioning or dif or computer adaptive testing or item bank or cross-cultural equivalence).ti,ab. (8081187)

MEDLINE

1 exp Schizophrenia/ or Schiz*.mp. (197059)

2 (PANSS* or "Positive and Negative Symptom" or "Positive and Negative Symptoms" or "Positive and Negative Syndrome" or "Positive and Negative Syndromes").mp. (9644)

3 exp Psychometrics/ or exp Outcome Assessment, Health Care/ or exp Observer Variation/ or exp Health Status Indicators/ or exp Reproducibility of Results/ or exp Discriminant Analysis/ or (instrumentation or methods).sh. or (validation study or comparative study).pt. or (clinimetr* or clinometr* or outcome measure* or agreement or precision or imprecision or precise values or repeatab* or ((replicab* or repeated) and (measure or measures or findings or result or results or test or tests))).mp. or (psychometr* or outcome assessment or observer variation or reproducib* or reliab* or unreliab* or valid* or coefficient of variation or coefficient or homogeneity or homogeneous or internal consistency or (cronbach* and (alpha or alphas)) or (item and (correlation* or selection* or reduction*)) or test-retest or (test and retest) or (reliab* and (test or retest)) or stability or interrater or inter-rater or intrarater or intra-rater or intertester or inter-tester or intratester or intra-tester or interobserver or inter-observer or intraobserver or intra-observer or intertechnician or inter-technician or intratechnician or intra-technician or interexaminer or inter-examiner or intraexaminer or intra-examiner or interassay or inter-assay or intraassay or intra-assay or interindividual or inter-individual or intraindividual or intra-individual or interparticipant or inter-participant or intraparticipant or intra-participant or kappa or kappas or generaliza* or generalisa* or concordance or (intraclass and correlation*) or discriminative or known group or factor analysis or factor analyses or factor structure or factor structures or dimension* or subscale* or (multitrait and scaling and (analysis or analyses)) or item discriminant or interscale correlation* or (error or errors) or individual variability or interval variability or rate variability or (variability and (analysis or values)) or (uncertainty and (measurement or measuring)) or standard error of measurement or sensitiv* or responsive* or (limit and detection) or minimal detectable concentration or interpretab* or ((minimal or minimally or clinical or clinically) and (important or significant or detectable) and (change or difference)) or (small* and (real or detectable) and (change or difference)) or meaningful change or ceiling effect or floor effect or item response model or IRT or Rasch or differential item functioning or DIF or computer adaptive testing or item bank or cross-cultural equivalence).tw. (9172531)
