# Supplementary material for: COSMIN systematic review and meta-analysis of the measurement properties of the Positive and Negative Syndrome Scale (PANSS)
Source: eClinicalMedicine. 2025 Apr 11;82:103155. doi: 10.1016/j.eclinm.2025.103155 (PMC12008685; doi:10.1016/j.eclinm.2025.103155)
Supplement: Appendix S4 [file mmc4.docx]

**Appendix 4.** Hypotheses defined by the review team

*Hypothesis 1:*

Between instruments measuring the same or a very similar construct, we expected a correlation of ≥0.5.

*Hypothesis 2:*

Between instruments measuring related but different constructs, we expected a correlation of ≤0.6.

*Hypothesis 3:*

Between instruments measuring dissimilar or contrary constructs, we expected a correlation of ≤0.4.

*Hypothesis 4:*

For responsiveness assessments calculating effect sizes from antipsychotic treatment trial data, we expected an effect size of 0.5±10%.
