# Supplementary material for: COSMIN systematic review and meta-analysis of the measurement properties of the Positive and Negative Syndrome Scale (PANSS)
Source: eClinicalMedicine. 2025 Apr 11;82:103155. doi: 10.1016/j.eclinm.2025.103155 (PMC12008685; doi:10.1016/j.eclinm.2025.103155)

**Appendix 7.** Meta-analytical results

Standard random-effects meta-analyses were conducted using Comprehensive Meta-Analysis Version 2; a) Cronbachs alphas for *internal consistency*, b) correlation coefficients for *cross-cultural validity,* c),d) and e) correlation coefficients for *reliability*, f) correlation coefficients for *criterion validity*, g) and h) correlation coefficients for *convergent validity.*

1. Cronbachs alphas for *internal consistency*


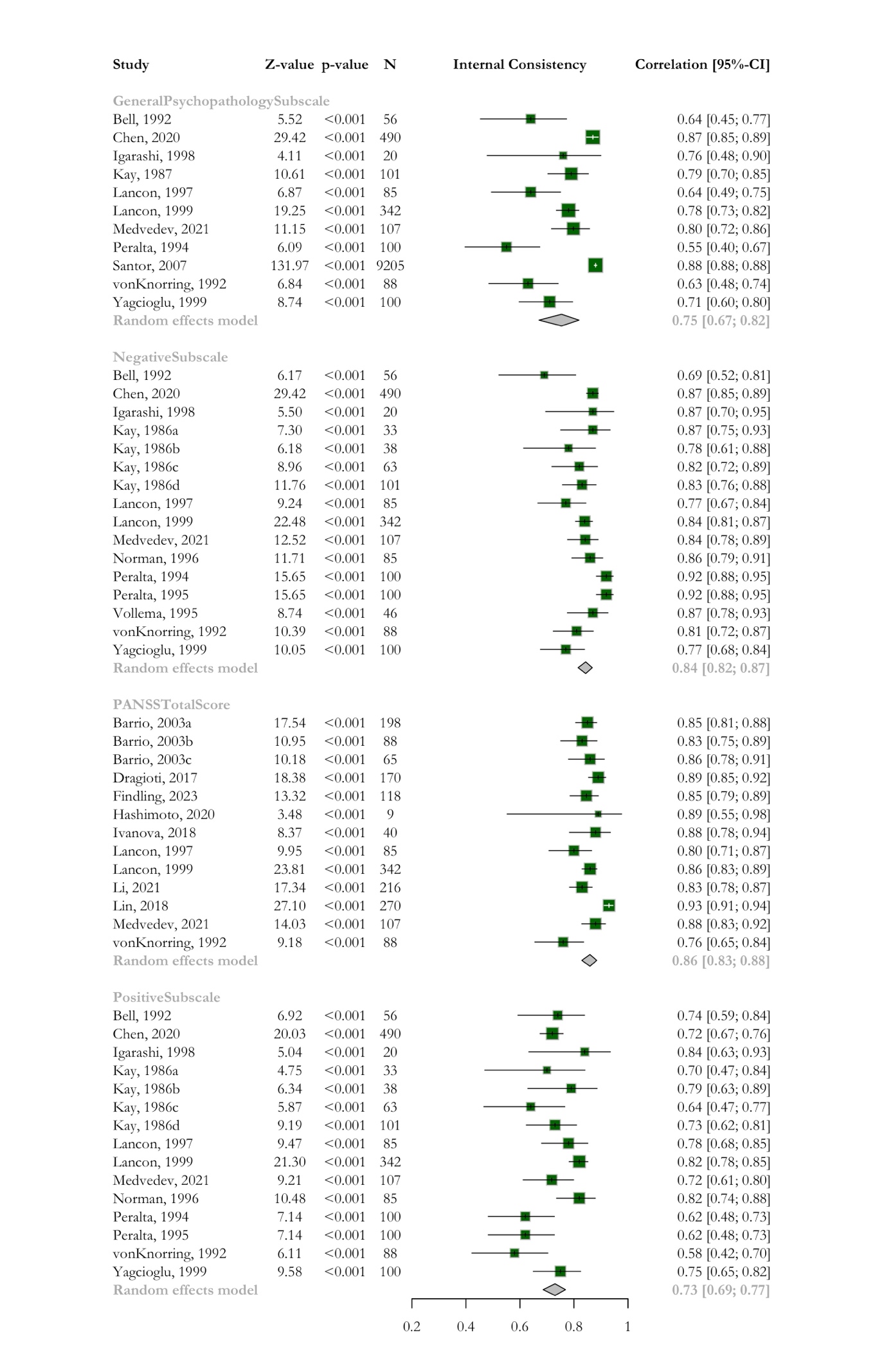


1. Correlation coefficients for *cross-cultural validity*


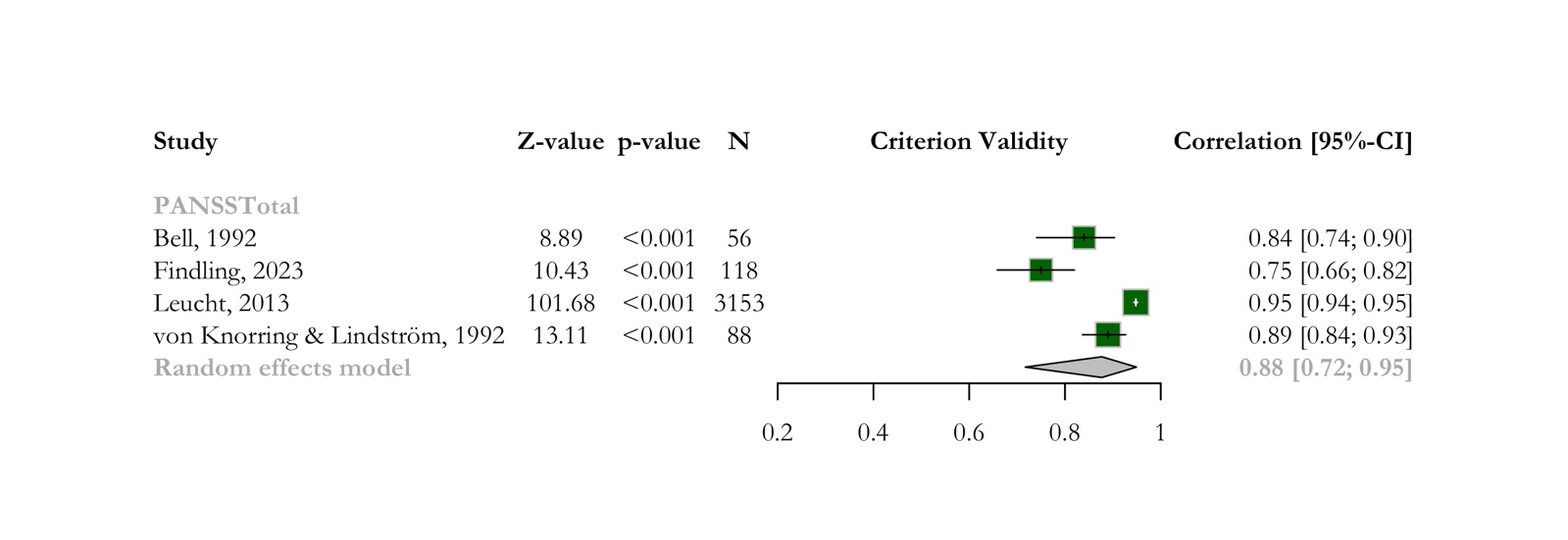


1. Correlation coefficients for *interrater reliability* (excluding studies with sample < 4)


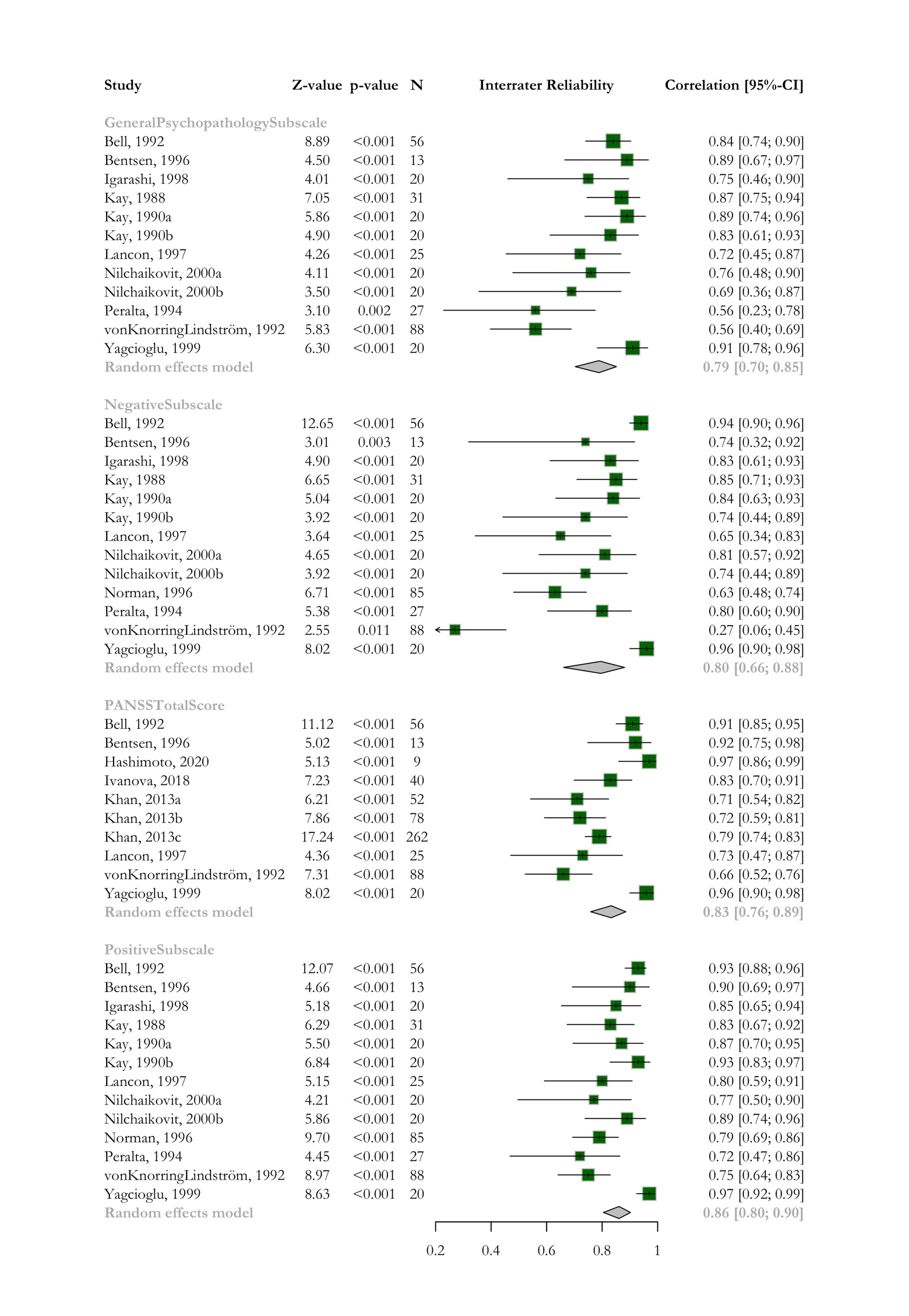


1. Correlation coefficients for *test-retest reliability*


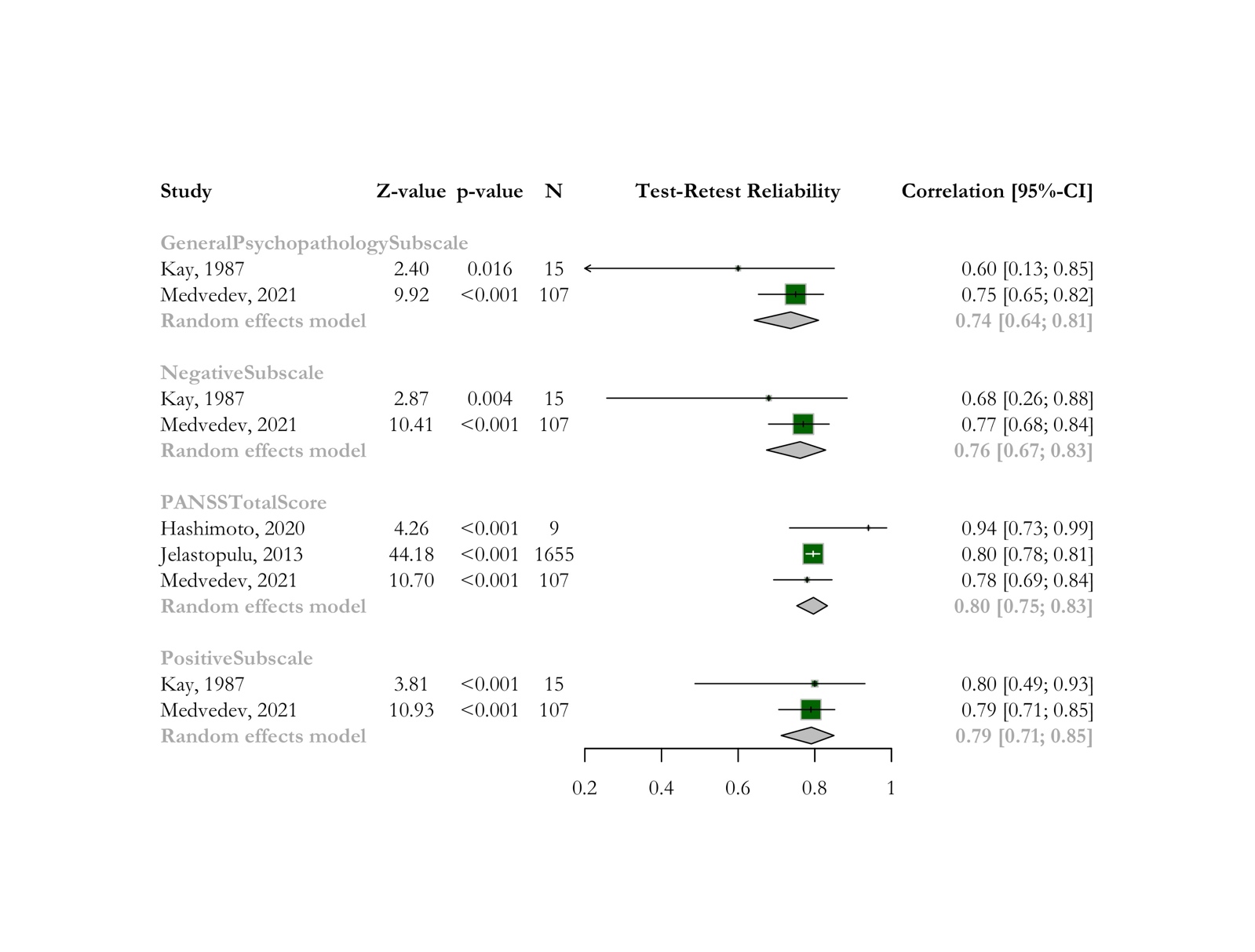


1. Correlation coefficients for *criterion validity*


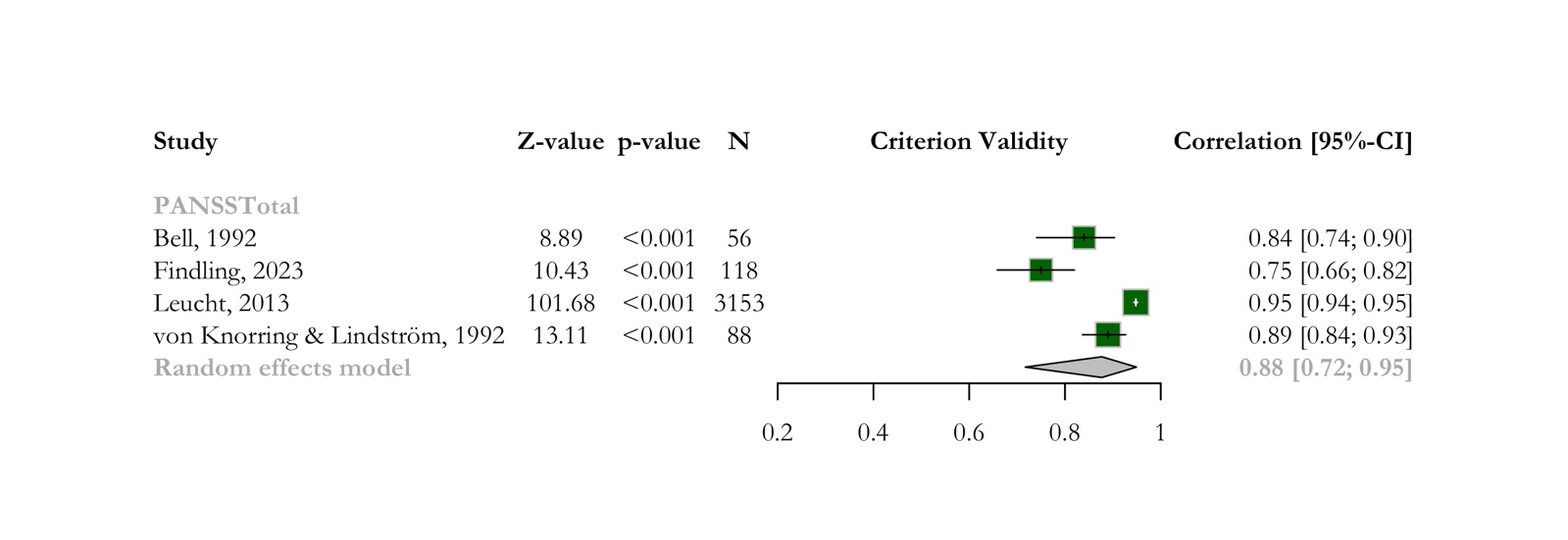


1. Correlation coefficients for *convergent validity*


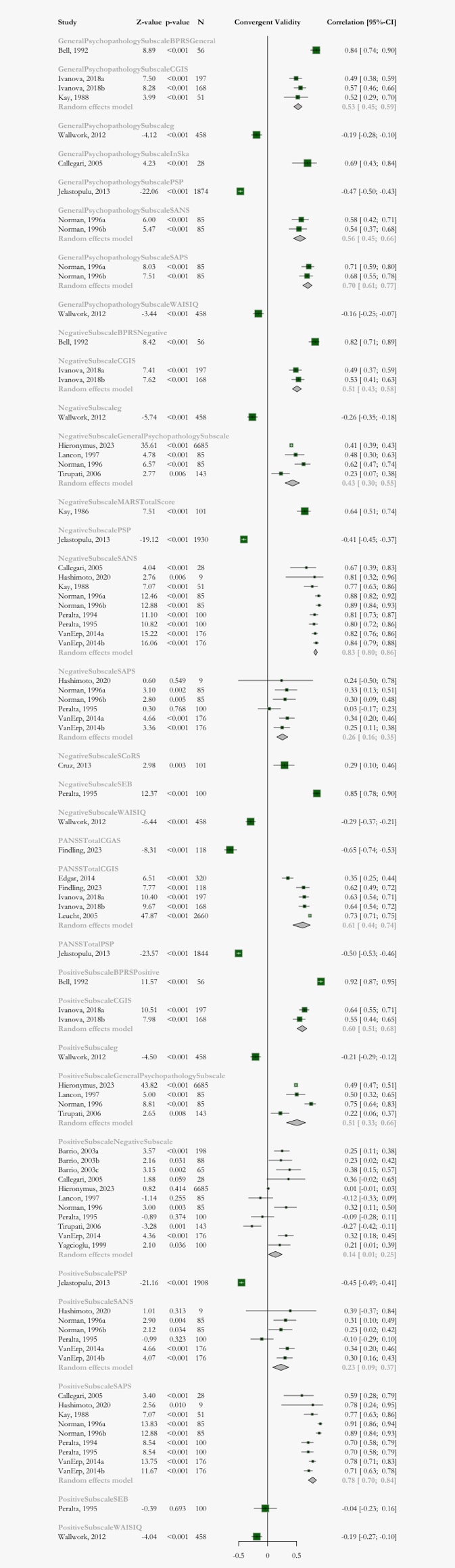

Supplement: Appendix S7 [file mmc7.docx]
