# Supplementary material for: COSMIN systematic review and meta-analysis of the measurement properties of the Positive and Negative Syndrome Scale (PANSS)
Source: eClinicalMedicine. 2025 Apr 11;82:103155. doi: 10.1016/j.eclinm.2025.103155 (PMC12008685; doi:10.1016/j.eclinm.2025.103155)
Supplement: Appendix S9 [file mmc9.docx]

| **Article** | **Method used** | **Finding(s) on the PANSS** |
| --- | --- | --- |
| Chen et al. (2020) Neurobiological Divergence of the Positive and Negative Schizophrenia Subtypes Identified on a New Factor Structure of Psychopathology Using Non-negative Factorization: An International Machine Learning Study. | Orthonormal projective non-negative matrix factorization (OPNMF) | - Four-factor structure (negative, positive, affective, and cognitive) has been identified |
| Esfahlani et al. (2017) Sensitivity of the Positive and Negative Syndrome Scale (PANSS) in Detecting Treatment Effects via Network Analysis. | Network analysis | **-** The PANSS is sensitive to dectecting antipsychotic treatment effects  - Interconnection of symptoms (PANSS items) appears to be different in treatment-responsive vs. treatment-resistant groups |

**Appendix 9.** PANSS assessments not implementable in the COSMIN framework
